# Poisoning of Pt/γ-Al_2_O_3_ Aqueous Phase Reforming Catalysts by Ketone and Diketone-Derived Surface Species

**DOI:** 10.1021/acscatal.3c04774

**Published:** 2024-01-16

**Authors:** Bryan J. Hare, Ricardo A. Garcia Carcamo, Luke L. Daemen, Yongqiang Cheng, Rachel B. Getman, Carsten Sievers

**Affiliations:** † School of Chemical & Biomolecular Engineering, Georgia Institute of Technology, Atlanta, Georgia 30332, United States; ‡ Department of Chemical and Biomolecular Engineering, Clemson University, Clemson, South Carolina 29634, United States; § Spallation Neutron Source, Oak Ridge National Laboratory, Oak Ridge, Tennessee 37830, United States

**Keywords:** di/ketones, deactivation, decarbonylation, spectroscopy, density functional theory

## Abstract

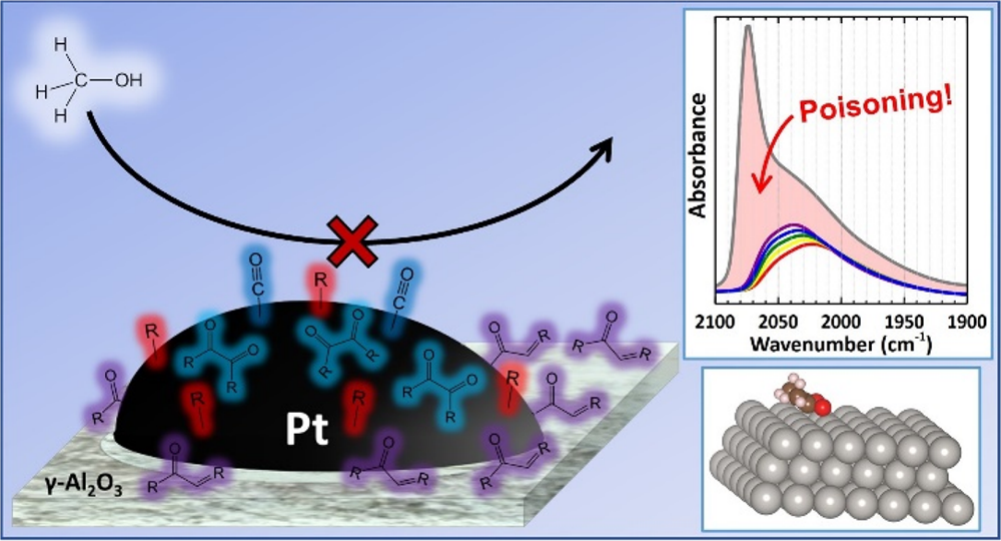

Strong adsorption of ketone and diketone byproducts and their fragmentation products during the aqueous phase reforming of biomass derived oxygenates is believed to be responsible for the deactivation of supported Pt catalysts. This study involves a combined experimental and theoretical approach to demonstrate the interactions of several model di/ketone poisons with Pt/γ-Al_2_O_3_ catalysts. Particular di/ketones were selected to reveal the effects of hydroxyl groups (acetone, hydroxyacetone), conjugation with C=C bonds (mesityl oxide), intramolecular distance between carbonyls in diketones (2,3-butanedione, 2,4-pentanedione), and length of terminal alkyl chains (3,4-hexanedione). The formation of adsorbed carbon monoxide (1900–2100 cm^–1^) as a decarbonylation product was probed using infrared spectroscopy and to calculate the extent of poisoning during subsequent methanol dehydrogenation based on the reduction of the ν(C≡O) band integral relative to experiments in which only methanol was dosed. Small Pt particles appeared less active in decarbonylation and were perhaps poisoned by strongly adsorbed di/ketones on undercoordinated metal sites and bulky conjugated species formed on the γ-Al_2_O_3_ support from aldol self-condensation. Larger Pt particles were more resistant to di/ketone poisoning due to higher decarbonylation activity yet still fell short of the expected yield of adsorbed CO from subsequent methanol activity. Vibrational spectra acquired using inelastic neutron scattering showed evidence for strongly binding methyl and acyl groups resulting from di/ketone decarbonylation on a Pt sponge at 250 °C. Adsorption energies and molecular configurations were obtained for di/ketones on a Pt(111) slab using density functional theory, revealing potential descriptors for predicting decarbonylation activity on highly coordinated metal sites. Calculated reaction energies suggest it is energetically favorable to reform surface methyl groups into adsorbed CO and H. However, the rate of this surface reaction is limited by a high activation barrier indicating that either improved APR catalyst designs or regeneration procedures may be necessary.

## Introduction

The drive toward a more sustainable future is contingent on the industrialization of renewable hydrogen. While it remains essential for several major production processes including those of fertilizers, electronics, and fuel, the vast majority of hydrogen is still acquired from fossil-based sources.^1−3^ This pressing concern continues to motivate research into the discovery and optimization of alternative methods for sustainable hydrogen production.

Biomass is an appealing source of hydrogen, and aqueous phase reforming (APR) is a heterogeneously catalyzed process that can extract this hydrogen from biomass-derived oxygenates dissolved in water.^4^ In principle, the conversion of C_*x*_H_2*x*+2_O_*x*_ feedstocks (glycerol, sorbitol, etc.) can result in high yields of H_2_ and CO_2_. This process is often conducted at temperatures of 180–250 °C, which is much lower than the temperatures required for pyrolysis and gasification but high enough to favor the water–gas shift.^5^ The gas products are easily separated from the bulk H_2_O phase, which can be recycled to reduce energy and material waste.

The transformation of oxygenates (polyols with a C:O ratio of 1:1) in liquid H_2_O was first studied by Dumesic et al. in 2002 while employing a Pt/Al_2_O_3_ catalyst.^4^ The authors put forth a wide array of possible reactions and chemical intermediates, given the acquired mixture of H_2_, CO_2_, and various alkanes. They proposed that the dehydrogenation → decarbonylation → water–gas shift reaction sequence is the most efficient for maximizing H_2_ formation. Since then, numerous catalysts have been designed and tested in APR to improve product yields and tailor the selectivity between H_2_ and alkanes.^6,7^ Today, it is widely acknowledged that supported Pt catalysts are most suitable for H_2_ production given the efficiency in C–H and C–C bond cleaving over this metal.^8,9^ This contrasts with other commonly utilized metals like Pd and Ni which also exhibit tendencies to break C–O bonds, thus resulting in alkane formation and decreased H_2_ yields.^10^ However, a severe decrease in APR activity is commonly observed while attempting to convert larger oxygenates, even on Pt catalysts.^4,11,12^ For the APR of polyols specifically, H_2_ yields follow the general trend of CH_3_OH > C_2_H_6_O_2_ > C_3_H_8_O_3_ > C_6_H_14_O_6_. This phenomenon is believed to originate from the formation of byproduct surface species that ultimately deactivate the catalyst.

There are recent studies by Davis et al. which focused on polyol oxidation in aqueous environments and the underlying causes of catalyst deactivation over supported Pt catalysts.^13,14^ It was shown that acetone, mesityl oxide (resulting from aldol condensation of acetone), and 2,4-pentanedione severely decreased the oxidation activity of the catalysts depending on the system pH. Because exposure to 2,4-pentanediol did not result in any decreases in conversion rates, it was deduced that ketone-based species were responsible for the deactivation of supported Pt catalysts through strong binding to the metal.

**Figure 1:**
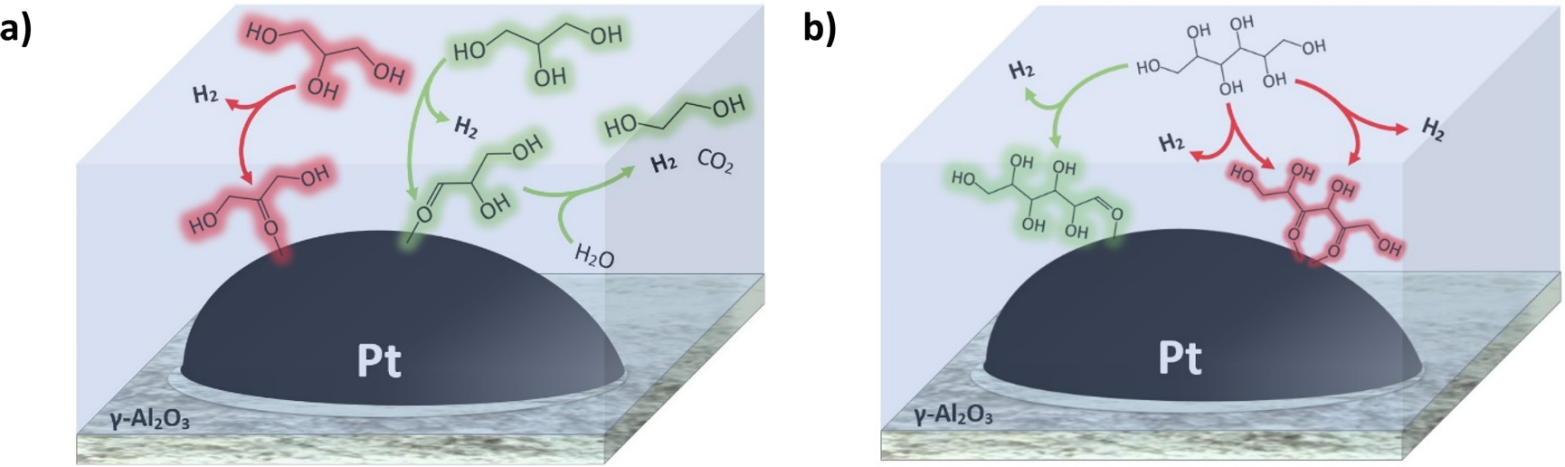
Illustrations of theorized catalyst poisons that may result from the nonselective adsorption and dehydrogenation of secondary alcohol groups on Pt/γ-Al_2_O_3_: **a)** ketone intermediate from glycerol and **b)** β-diketone intermediate from sorbitol.

The mechanism for the APR of larger oxygenates is particularly complicated, given the presence of numerous functional groups and possible side reactions. However, it is believed that the reaction pathway for H_2_ and CO_2_ production involves the decarbonylation of aldehyde intermediates formed from the dehydrogenation of primary (1°) alcohol groups.^15−17^ Alternatively, ketone species may be formed in APR by the nonselective adsorption and dehydrogenation of secondary (2°) alcohol groups found on larger oxygenates starting with glycerol (Figure 1a). Ketones are expected to decarbonylate less readily given increased steric hindrance.^18−20^ The formation of these species is more likely to happen with larger oxygenates with higher 2°:1° alcohol group ratios.

Furthermore, larger polyol or sugar reagents could even form diketone species (Figure 1b). For oxygenates as sizable as sorbitol, these diketone species could potentially exhibit the additional complication of varying the proximities between the carbonyl groups. They can be adjacent (α diketone) or split by one or two carbon atoms (β and γ diketones, respectively). While interactions of simple ketones, such as acetone, with metal surfaces have been extensively probed, similar studies with diketones are seldom conducted, although it is known that simultaneous surface interactions of multiple functional groups can play a significant role in catalytic conversion of oxygenates.^21^

Herein, we use infrared (IR) spectroscopy and inelastic neutron scattering (INS) coupled with density functional theory (DFT) to study the adsorption, reaction, and poisoning of Pt/γ-Al_2_O_3_ catalysts by ketones and diketones. Catalyst poisoning by strong-binding di/ketones and their methyl and acyl fragments is discussed. While potential Pt poisoning surface species have been identified and understanding of di/ketone decarbonylation has been established, perspectives are provided for improving Pt catalyst durability and longevity in APR applications.

## Methods

### Materials

A 1% Pt/γ-Al_2_O_3_ catalyst with an average Pt particle size of 1.1 nm (σ = 0.4 nm), measured with transmission electron micrograms, was synthesized via wet impregnation with a H_2_PtCl_6_ (Sigma-Aldrich ≥99.9% trace metal basis) precursor and γ-Al_2_O_3_ (Alfa Aesar 99.97%). This was intentionally made to possess smaller particles and is referred to as Pt_S_/γ-Al_2_O_3_. For comparison, a commercially obtained 5% Pt/γ-Al_2_O_3_ catalyst (Sigma-Aldrich #205974) with an average Pt particle size of ∼4.6 nm (σ = 1.2 nm) was used. This sample is termed Pt_L_/γ-Al_2_O_3_ given its larger metal particle size. The (Brunauer–Emmett–Teller) BET surface areas of both catalysts along with the Lewis acidity of the γ-Al_2_O_3_ were previously calculated through N_2_ and pyridine adsorption, respectively.^22^ Catalysts were reduced at 500 °C in 7% (v/v) H_2_/He for 2 h prior to experiments. While this temperature is sufficient for reducing supported Pt particles, the low concentration of H_2_ was used to limit the extent of sintering.^22^ A variety of organic reagents suspected of poisoning Pt were used herein (Table 1).

**Table 1:**
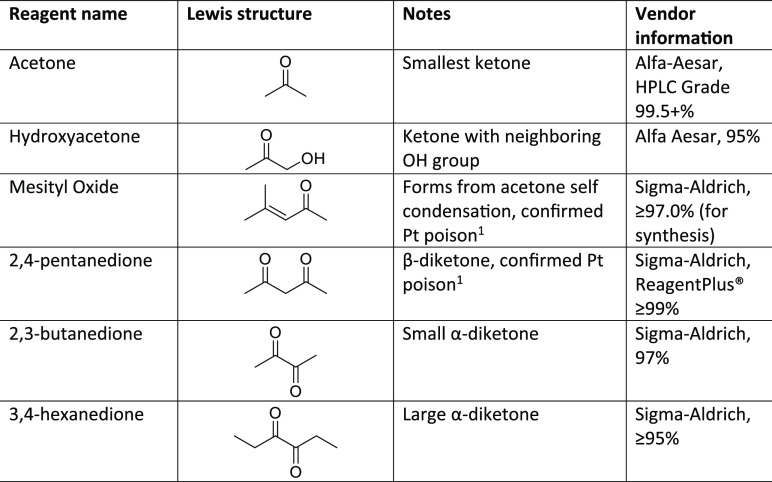
Ketone and Diketone Reagents Used

a A superscript 1 denotes the following: from refs (13 and 14).

### Infrared Spectroscopy

Catalyst powders were hydraulically pressed into self-supporting wafers that were positioned within a high vacuum (<4.5 × 10^–7^ mbar) chamber with ZnSe windows. IR spectra were acquired using a Thermo Scientific Nicolet 8700 FT-IR spectrometer and analyzed with Thermo Scientific Omnic software. Each spectrum was an average of 64 scans collected with a resolution of 1.928 cm^–1^, an optical velocity of 1.8988 nm, and an aperture of 75. Wafers were activated under high vacuum at 450 °C (10 °C/min) for 1 h.

**Figure 2:**
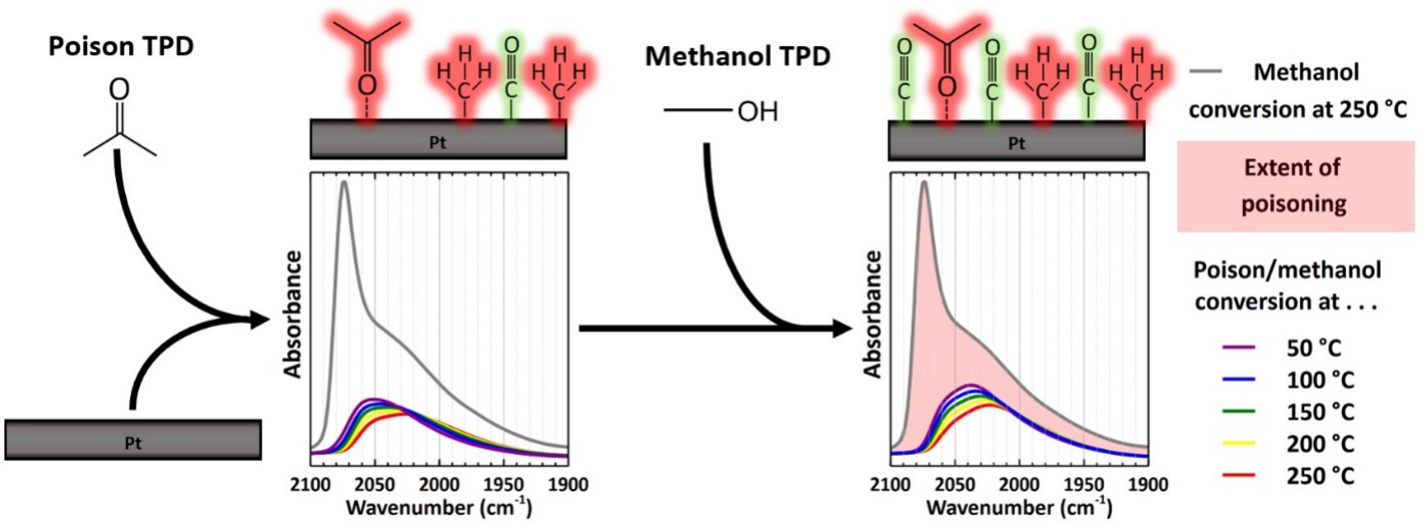
Sequence of TPDs with poisoning species and methanol as observed by IR spectroscopy.

Each experiment consisted of two sequential temperature-programmed desorption steps (TPD) under a high vacuum (Figure 2). The activated catalyst wafers were first exposed to 0.5 mbar of the poisoning di/ketone vapor for 10 min at 50 °C. Following evacuation of the chamber, the temperature was increased to 100, 150, 200, and 250 °C (10 °C/min) to observe the decarbonylation, or lack thereof, of adsorbed di/ketones. The poisoned wafer was then exposed to 0.5 mbar of methanol (VWR International ≥99.8%) vapor for 15 min. Methanol is a suitable reagent for this study as it readily undergoes dehydrogenation, a crucial step of APR, on Pt and concomitantly forms adsorbed carbon monoxide, which maintains a strong presence in the IR spectrum. The chamber was once again evacuated, and the aforementioned TPD was repeated.

The resulting IR bands of adsorbed CO were integrated to assess which metal sites facilitated di/ketone decarbonylation and/or methanol dehydrogenation. A semiquantitative correlation exists between the IR band integrals and the conversion of di/ketones and methanol. IR band integrals were normalized with respect to the estimated amount of available Pt sites calculated using the Pt/γ-Al_2_O_3_ wafer mass and Pt dispersion. The Pt dispersion of each catalyst was estimated using eq 1 initially presented by Bergeret and Gallezot.^23^
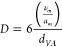
1The dispersion (*D*) for each catalyst was calculated by using the measured average Pt particle sizes (*d*_*VA*_). Values of 15.1 Å^3^ and 8.07 Å^2^ were used for the Pt atomic bulk metal volume (*v*_*m*_) and the Pt surface atom area (*a*_*m*_), respectively.^23^

### Inelastic Neutron Scattering

A Pt sponge (Sigma-Aldrich, ≥99.9% trace metal basis) was employed for inelastic neutron scattering (INS) experiments to isolate and observe metal-bound surface species. The sponge was first reduced with 10% (v/v) H_2_/He at 250 °C for 1 h. BET isotherms^24^ were collected via N_2_ physisorption using a Micromeritics Gemini VII analyzer after degassing at 250 °C for 2 h. Based on the measured ∼36 m^2^/g surface area, 1.5 g of the Pt sponge was packed into an aluminum vessel within a dry helium glovebox. Under HV at 250 °C, the sponge was exposed to small amounts of di/ketone vapor (∼0.5 mbar) for 15 min. The vessel was then again evacuated to remove any physisorbed species.

The VISION vibrational spectrometer at the Oak Ridge National Laboratory (ORNL) Spallation Neutron Source (SNS)^25^ was used to perform INS experiments and observe the vibrational modes of Pt-adsorbed surface species at low energies (<500 cm^–1^). The instrument determined the incident neutron energy with time-of-flight, while the final neutron energy was fixed by Bragg reflection on a series of 13 curved, pyrolytic graphite analyzers. Parameters included a dynamic range of 0–1000 meV, resolution of <1.0–1.5%, and a diffraction Q range of 1.5–30 Å^–1^. The beamline was equipped with a closed-cycle, top-loading refrigerator maintained at 5–600 K. Data was collected in event mode and subsequently refined with background subtraction (clean Pt sample and aluminum vessel), rebinning to improve count statistics, and some smoothing (moving average) to reduce statistical noise.

### Computational Methods

Molecule adsorption energies were calculated according to eq 2, subtracting the electronic energy of the clean Pt surface (*E*_*slab*_) and the gas phase molecule (*E*_*Adsorbate*_) from the electronic energy of the adsorbed molecule (*E*_Adsorbate*_).
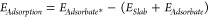
2Reaction energies (*E*_*rxn*_) were calculated by subtracting the electronic energies of the reactants (*E*_*Reactants,j*_) from the electronic energies of the products (*E*_*Products,i*_), as shown in eq 3.
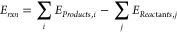
3

Pt surfaces were modeled with periodic Pt(111) slabs, constructed from the calculated structure of bulk fcc Pt. Three types of unit cells were used; one larger cell used to compute binding energies and reaction energies (given these calculations involve larger adsorbates), one smaller and more computationally tractable cell for computing neutron vibrational spectra of methyl and acyl surface species, and one cell for calculating gas phase molecules.

Calculations for binding energies and reaction energies employed three-layer-thick 6 Pt × 6 Pt slabs in a supercell with dimensions of a = b = 16.8 Å, c = 19.6 Å, α = β = 90°, and γ = 60° (Figure S1). A three-layer model was used to make calculations with these larger adsorbates computationally tractable. The results in Table S1 show only minor differences (0.08 eV) in the calculated adsorbate binding energies from adding a fourth metal layer. A maximum of one adsorbate was included on each slab model, and the closest distance between adsorbates in neighboring periodic images was 11 Å. These configurations were tested on top, fcc, hcp, and bridge sites. The configuration and site that gave the lowest energy is the one reported in the manuscript.

Calculations for neutron vibrational spectra employed a four-layer-thick 2 Pt × 4 Pt Pt(111) slab with one methyl (or acyl) group attached directly to a Pt atom on the surface (Figure S2). As this cell is smaller, the fourth metal layer can be used without significantly impacting the computational expense. The dimensions of this cell were a = 5.5 Å, b = 9.6 Å, and c = 16.8 Å and α = β = γ = 90°. Calculations for gas phase molecules employed a unit cell with lengths of 20 Å × 20.1 Å × 20.2 Å and only one gas phase species per unit cell.

DFT calculations were performed using the Vienna Ab initio Simulation Package (VASP).^26−30^ Plane waves were included up to energy cutoffs of 400 eV for calculation of adsorption and reaction energies and 600 eV for neutron vibrational spectra. All DFT calculations employed PAW pseudopotentials^31,32^ and the PBE exchange correlation functional. Calculations of binding and reaction energies additionally employed D3 dispersion corrections^33^ with Becke-Johnson damping.^34,35^ Spin polarization was turned on, and dipole corrections were applied in the direction normal to the surface. Calculations of neutron vibrational spectra employed relativistic effects and spin–orbit coupling. Gamma-centered Monkhorst–Pack^36^ k-point meshes were used in all DFT calculations. Meshes of 3 × 3 × 1 and 6 × 3 × 1 were employed for the 6 Pt × 6 Pt and 2 Pt × 4 Pt slabs, respectively, and meshes of 1 × 1 × 1 were used for gas molecule calculations. A test using a higher k-point mesh is provided in Table S2 showing that using a higher k-point mesh has minimal effects (0.03 eV difference) on the calculated adsorbate binding energies. Electronic structures were considered to be converged when the difference in energy between subsequent iterations was no larger than 10^–6^ eV for adsorption and reaction energy calculations and 10^–8^ eV for neutron vibrational spectra calculations. Geometries were considered to be converged when the forces on all atoms in the supercell were no larger than 0.03 eV/Å for adsorption and reaction energy calculations and 0.002 eV/Å for neutron vibrational spectra calculations.

Neutron vibrational spectra were computed from the interatomic force constants using the finite displacement method on the calculated electronic structures. Vibrational eigenfrequencies were then calculated using Phonopy.^37^ The OCLIMAX software was used to convert the DFT-calculated phonon results to the simulated VISION spectra.^38^ It first calculates fundamentals and overtones and combination bands. Then, it convolutes the instrument resolution function with the calculated vibrational spectrum and presents vibrational modes that are too close to being resolved as a single band.

## Results

### Characterization

The metal particle size distribution of Pt_S_/γ-Al_2_O_3_ ranged from ∼0.5 to ∼2.0 nm with an average diameter of ∼1.1 nm. That of Pt_L_/γ-Al_2_O_3_ was ∼1.0 to ∼8.3 nm with an average diameter of ∼4.6 nm. Therefore, the former catalyst is expected to have a high fraction of undercoordinated Pt sites, while the latter is expected to have a high fraction of terrace sites. The Pt dispersion of Pt_S_/γ-Al_2_O_3_ and Pt_L_/γ-Al_2_O_3_ was estimated to be about 100% and 24%, respectively. Both catalysts exhibited similar BET surface areas of ∼70 m^2^/g. The concentration of Lewis acid sites on the γ-Al_2_O_3_ that can retain pyridine at concentrations of 150 and 250 °C was measured as 104 and 72 μmol/g, respectively.

### Di/Ketone Adsorption on Small Pt Particles

**Figure 3:**
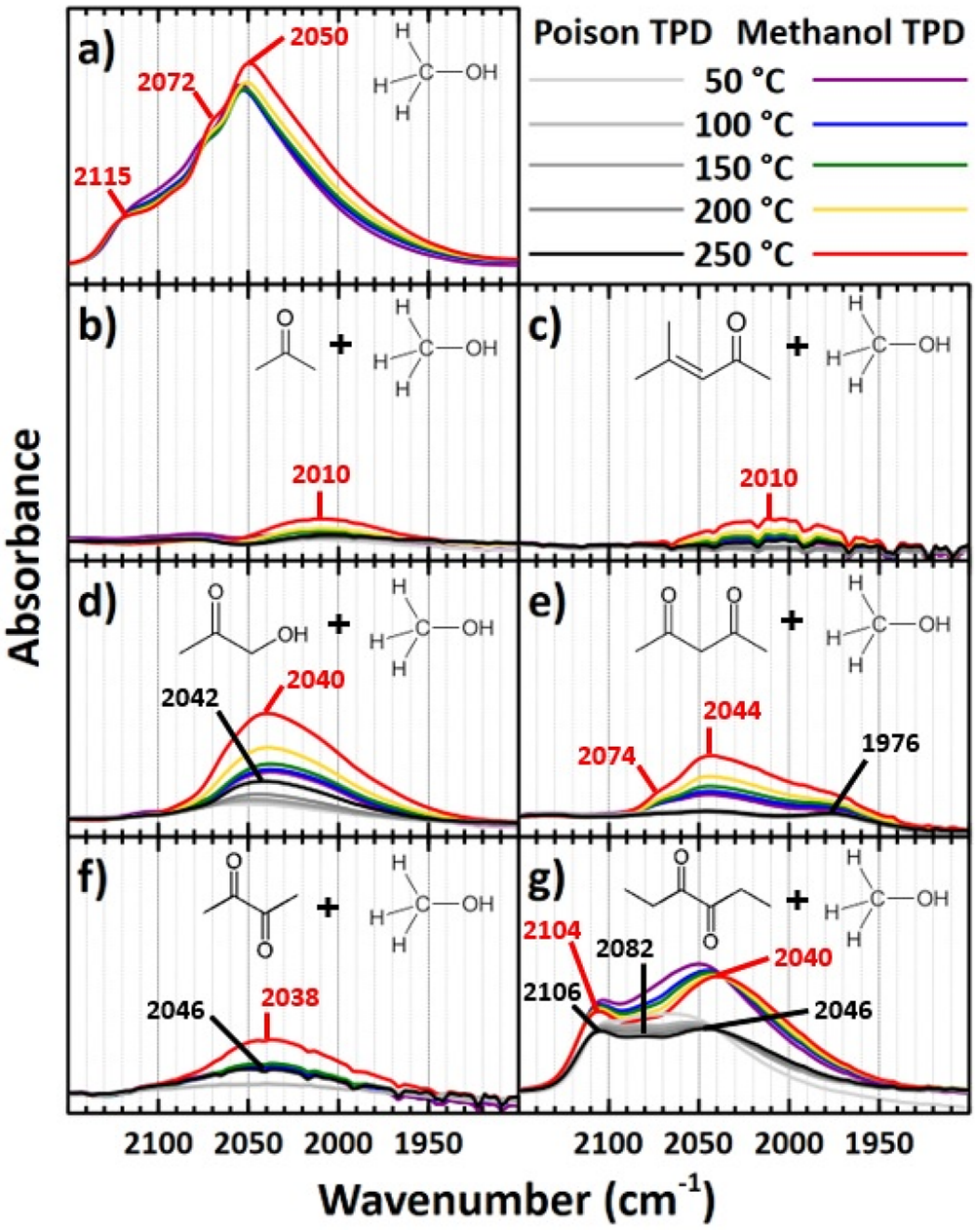
IR spectra of adsorbed CO during TPD experiments with Pt_S_/γ-Al_2_O_3_ (∼1.1 nm Pt particles) up to 250 °C. **a)** Methanol TPD on clean Pt_S_/γ-Al_2_O_3_. Methanol TPDs follow poison TPDs in which the poison is **b)** acetone, **c)** mesityl oxide, **d)** hydroxyacetone, **e)** 2,4-pentanedione, **f)** 2,3-butanedione, and **g)** 3,4-hexanedione. Major band frequencies are labeled for adsorbed CO resulting from poison decarbonylation (black) and methanol dehydrogenation (red). All scans were taken at 50 °C.

The conversion of methanol on Pt/γ-Al_2_O_3_ catalysts is known to result in adsorbed CO due to dehydrogenation on metal sites.^22^ This is evident by band developments in the 1900–2150 and 1750–1900 cm^–1^ regions that are attributed to the stretching modes of linear CO (CO_L_) and bridging CO (CO_B_), respectively.^39,40^ During methanol conversion on Pt_S_/γ-Al_2_O_3_, the primary ν(C≡O) band was centered at 2050 cm^–1^ and assigned to CO_L_ on metallic Pt sites (Figure 3a). The large, low frequency shoulder that extended as low as 1900 cm^–1^ is characteristic of CO adsorbed near the interface between metal particles and Lewis acidic supports such as γ-Al_2_O_3_.^41,42^ The midfrequency shoulder at 2072 cm^–1^ was attributed to adsorbed CO that is closely surrounded by other CO species that engage in dipole–dipole coupling, a phenomenon that results in slightly stronger C≡O bonds and a distinguishable band.^43^ The highest frequency shoulder at 2115 cm^–1^ is often assigned to CO adsorbed to single Pt atoms^44,45^ or Pt sites near adsorbed Cl^46^ from the H_2_PtCl_6_ synthesis precursor. The specific IR band assignments are also illustrated in Figure S3.

This deconvolution of the CO stretching bands provided insight into the locations of the CO formed from the decarbonylation of ketones and diketones on Pt_S_/γ-Al_2_O_3_ (Figure 3). In addition, the ν(C≡O) frequencies observed during subsequent methanol dehydrogenation showed which sites are occupied by adsorbed poisons. Slight frequency shifts for individual ν(CO) bands observed with increasing temperature, if any, were attributed to increased dipole–dipole coupling between adjacent CO species formed at higher di/ketone and methanol conversion, migration of CO to different sites, variations of Pt particle electron density due to changes in coverage by electron donating and withdrawing surface species, or a combination thereof.^46−48^ The full IR spectra of products from methanol and di/ketones adsorbed to Pt_S_/γ-Al_2_O_3_ can be seen in Figure S4.

On small Pt particles, no decarbonylation activity was observed when acetone and mesityl oxide were adsorbed (Figures 3b and 3c). The development of the CO_L_ band during subsequent methanol adsorption was very similar for these ketone species given both sets of spectra include a small broad band centered at 2010 cm^–1^. This could be, in part, due to the conversion of acetone into mesityl oxide by aldol condensation on the Lewis acid sites of the γ-Al_2_O_3_ support,^49,50^ which creates a similar environment for CO species adsorbed on Pt. Increasing the temperature up to 250 °C resulted in only a slight increase in intensity of the 2010 cm^–1^ band, while the broadness remained and made it difficult to track frequency shifts, if any. As hydroxyacetone was adsorbed, a small CO_L_ band appeared at 2042 cm^–1^ evident of some decarbonylation on Pt particles (Figure 3d). The further notable development of this band during methanol exposure suggests methanol was still able to dehydrogenate on open Pt sites, including those near the metal/support interface. However, the high frequency bands at 2072 and 2115 cm^–1^ seen during methanol adsorption on clean Pt_S_/γ-Al_2_O_3_ were absent.

There was minimal decarbonylation of 2,4-pentanedione, the β-diketone, even as high as 250 °C (Figure 3e). A very small band at 1976 cm^–1^ suggested the only decarbonylation occurred near the metal/support interface. However, when methanol was adsorbed, the appearance of distinct bands at 2044 and 2074 cm^–1^ and a low frequency shoulder implied that methanol was still able to dehydrogenate on a range of different metal sites. Yet, the magnitude of the band was significantly reduced compared to that of the control experiment. The α-diketones, 2,3-butanedione and 3,4-hexanedione, behaved very differently on small Pt particles (Figures 3f and 3g). Adsorption of the former species resulted in a small, lone broad band at 2046 cm^–1^. During exposure to methanol, this band and a low frequency shoulder grew only slightly with the primary frequency red shifting to 2038 cm^–1^. This observation was heavily contrasted by that of 3,4-hexanedione adsorption. Not only did the larger α-diketone decarbonylate on a variety of sites given the distinct bands observed at 2046, 2082, and 2106 cm^–1^, but the resulting CO_L_ band was notably larger than that seen during 2,3-butanedione adsorption. This suggested that 3,4-hexanedione decarbonylated on metal sites much more readily than 2,3-butanedione. Subsequent methanol adsorption on 3,4-hexanedione-poisoned Pt_S_/γ-Al_2_O_3_ led to growth of the 2046 and 2106 cm^–1^ bands and a gradual shift to 2050 and 2104 cm^–1^, respectively, at 250 °C. The low frequency shoulder also grew in magnitude. Because the CO_L_ band within this particular experiment somewhat resembles the shape and frequencies of those of the control experiment, it may be inferred that the formation of CO on the 3,4-hexanedione-poisoned Pt_S_/γ-Al_2_O_3_ catalyst is not site specific. In other words, coverage by CO and other adsorbates resulting from 3,4-hexanedione decarbonylation on the Pt surface is well mixed. However, the total integral of the final CO_L_ band during exposure to 3,4-hexanedione and methanol also fell short of that of the control experiment.

### Di/Ketone Adsorption on Large Pt Particles

**Figure 4:**
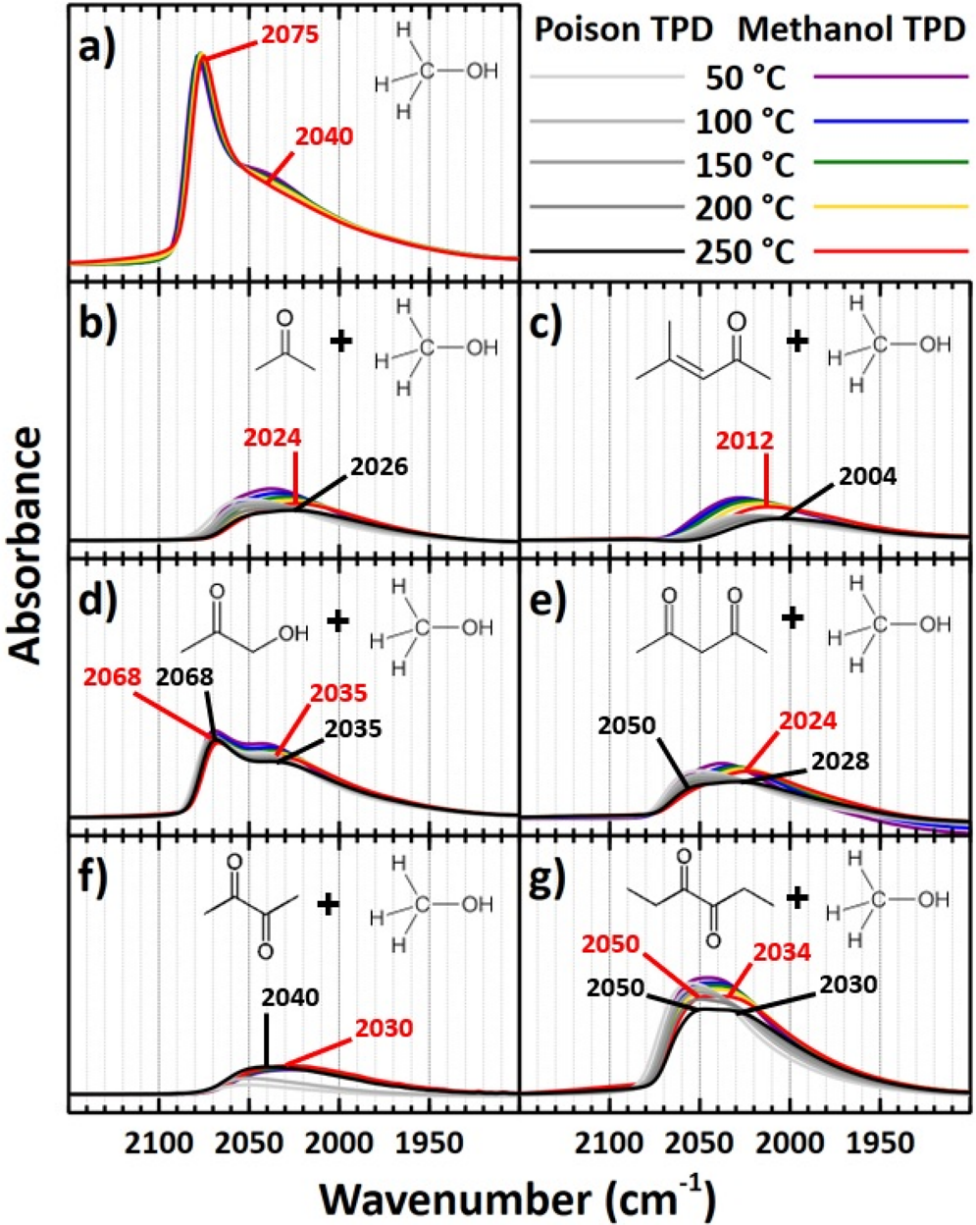
IR spectra of adsorbed CO during TPD experiments with Pt_L_/γ-Al_2_O_3_ (∼4.6 nm Pt particles) up to 250 °C. **a)** Methanol TPD on clean Pt_L_/γ-Al_2_O_3_. Methanol TPDs following poison TPDs in which the poison is **b)** acetone, **c)** mesityl oxide, **d)** hydroxyacetone, **e)** 2,4-pentanedione, **f)** 2,3-butanedione, and **g)** 3,4-hexanedione. Major band frequencies are labeled for adsorbed CO resulting from poison decarbonylation (black) and methanol dehydrogenation (red). All scans were performed at 50 °C.

The conversion of methanol on the Pt_L_/γ-Al_2_O_3_ catalyst also resulted in dehydrogenation as indicated by a strong ν(C≡O) band associated with CO_L_ (Figure 4a). However, this band exhibited a shape different from that of CO_L_ on smaller Pt particles. For instance, the contribution at 2075 cm^–1^ was dominant for CO on large Pt particles. We previously attributed this to adsorbed CO species participating in dipole–dipole coupling, a phenomenon that occurs to a much greater extent on the larger terraces of larger Pt particles.^51^ The spectra also contained a smaller feature centered at 2040 cm^–1^ and a broad low frequency shoulder that extended as low as 1900 cm^–1^. Similar to that of CO on small Pt particles, these represent CO_L_ bound in isolation to metallic Pt and Pt near the metal/support interface, respectively.^41,42^ Full IR spectra of adsorbed di/ketones on Pt_L_/γ-Al_2_O_3_ are displayed in Figure S5.

Acetone and mesityl oxide conversion on Pt_L_/γ-Al_2_O_3_ resulted in similar CO_L_ bands (Figures 4b and 4c), due to decarbonylation, with frequencies of 2026 and 2004 cm^–1^, respectively. Although small and broad, these bands appeared larger than those on Pt_S_/γ-Al_2_O_3_, suggesting that a greater extent of decarbonylation took place on larger Pt particles or highly coordinated Pt sites. Yet, only slight growth of the CO_L_ band occurred during extended methanol exposure. In addition, there was an apparent red shift and broadening of the CO_L_ bands as the temperature increased from 50 to 250 °C during experiments with acetone and mesityl oxide. Specifically, during the conversion of acetone, the ν(C≡O) frequency shifted from 2052 to 2026 cm^–1^. During subsequent methanol adsorption at 50 °C, the band grew slightly with a new primary ν(C≡O) frequency of 2038 cm^–1^, which red-shifted similarly to 2024 cm^–1^ upon heating to 250 °C. For the mesityl oxide experiment, the shifts were 2028 to 2004 cm^–1^ and 2028 to 2012 cm^–1^ after dosing mesityl oxide and methanol, respectively. These observations suggest that while ketone decarbonylation and methanol dehydrogenation initially take place on highly coordinated sites, the red shifts likely represent the migration of adsorbed CO to more undercoordinated sites, where it binds more strongly,^52^ with increasing temperature. The CO_L_ band for hydroxyacetone adsorption on Pt_L_/γ-Al_2_O_3_ also appeared much larger than that on Pt_S_/γ-Al_2_O_3_ and exhibited two distinct features (Figure 4d). A sharp band is at 2068 cm^–1^, and a broad band is centered at 2035 cm^–1^. The existence of these 2068 and 2035 cm^–1^ bands implied that hydroxyacetone decarbonylates on a variety of metal sites such as those with high and low coordination, respectively.^48^ Minimal growth of these bands occurred during subsequent methanol conversion. In fact, the bands exhibited consistent frequencies at 2035 and 2068 cm^–1^ from hydroxyacetone conversion at 50 °C to incomplete methanol dehydrogenation at 250 °C, suggesting that the existing surface species were stable on large Pt particles.

When adsorbed to Pt_L_/γ-Al_2_O_3_, 2,4-pentanedione decarbonylated to a limited extent to form a small CO_L_ band with distinct features at 2050 and 2028 cm^–1^ (Figure 4e). Given the lack of band growth during the respective TPD, methanol dehydrogenation was severely hindered on the 2,4-pentanedione-poisoned surface. Like those of acetone and mesityl oxide, the spectra revealed a red shift of the overall band with increasing temperatures, suggesting adsorbed CO migrated to undercoordinated Pt sites.^48^ Adsorption of the α-diketones displayed contrasting effects (Figures 4f and 4g) on Pt_L_/γ-Al_2_O_3_, like those seen on Pt_S_/γ-Al_2_O_3_. 2,3-Butanedione was decarbonylated to a minimal extent even at 250 °C, leading to a weak, broad CO_L_ band centered at 2040 cm^–1^ with a low frequency shoulder. Subsequent dosing of methanol resulted in virtually no changes to the CO_L_ band with the exception of a red shift to 2030 cm^–1^_,_ suggesting that molecular 2,3-butanedione or a derivative surface species bound very strongly to the Pt particles and blocked sites that would otherwise be available for methanol dehydrogenation. On the contrary, 3,4-hexanedione was readily decarbonylated as it did on small Pt particles, with distinct CO_L_ bands at 2050 and 2030 cm^–1^. Slight growth of these bands occurred following methanol adsorption, with only a slight shift of the latter band to 2034 cm^–1^.

### Evolution of CO_L_ Band Integrals

Quantitative analysis of the overall CO_L_ band was conducted to estimate the extent of poisoning by each ketone and diketone species. The CO_L_ integrals acquired during poison and methanol adsorption were compared directly to that of methanol conversion on clean Pt/γ-Al_2_O_3_ catalysts at the same temperatures from 50 to 250 °C (Figure S6).

The focus was on the fractional differences in CO_L_ band integrals at 250 °C following methanol adsorption (Table 2) given typical APR temperatures fall in the range of 200–270 °C.^6^ Overall, greater reductions in CO_L_ band integrals were observed during methanol conversion on Pt_S_/γ-Al_2_O_3_ when pre-exposed to di/ketones in comparison to those observed while using Pt_L_/γ-Al_2_O_3_. This may suggest that larger Pt particles, or highly coordinated Pt sites, are more resistant to poisoning by di- or ketones or fragments from their decarbonylation. Regardless of the Pt particle size, the least severe poisons were 3,4-hexanedione and hydroxyacetone.

**Table 2:**
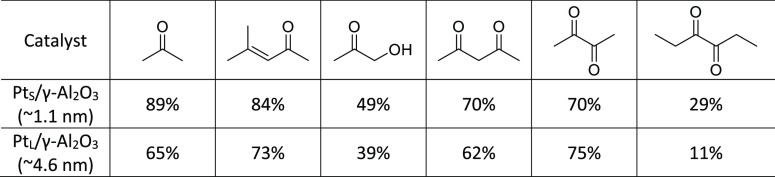
Fractional Decreases in Total CO_L_ Band Integrals at 250 °C Following Poison and Methanol TPDs on Pt/γ-Al_2_O_3_ Catalysts under HV^a^

a The calculated range of error was ±2.4%.

For Pt_S_/γ-Al_2_O_3_, preadsorbed acetone resulted in the highest reduction of the CO_L_ band integral during methanol conversion at 250 °C (89%). Preadsorbed mesityl oxide showed a similar effect with a 84% reduction of the CO_L_ band integral. Because acetone readily converts into mesityl oxide through aldol condensation on γ-Al_2_O_3_,^49,53^ it is possible that smaller Pt particles, which consist of many interfacial sites, are also poisoned by species formed from acid-catalyzed reactions on the support. A previous IR spectroscopy study revealed that all of the di/ketones in this study engage in acid–base reactions when adsorbed to γ-Al_2_O_3_, most of which involved the formation of heavier conjugated surface species from aldol condensation at 250 °C.^54^

For Pt_L_/γ-Al_2_O_3_, preadsorbed 2,3-butanedione led to the greatest CO_L_ band integral reduction (75%), suggesting this particular diketone acts as a strong binding poison. A similar magnitude was observed with mesityl oxide (73%), suggesting that both interfacial sites and more metallic coordinated sites are susceptible to strong binding by the conjugated ketone.

In a separate experiment, the CO_L_ band integral was monitored for 10 min during methanol dehydrogenation on both clean and 2,3-butanedione-poisoned Pt_L_/γ-Al_2_O_3_ at 150 °C (Figure S7). On clean Pt_L_/γ-Al_2_O_3_, the CO formation rate was very high (∼14.7 au/min) for the first minute before the CO_L_ band integral began saturating at ∼13 au. While the initial CO_L_ band integral for 2,3-butanedione-poisoned Pt_L_/γ-Al_2_O_3_ was ∼5 au, the CO formation rate during subsequent methanol dehydrogenation was severely reduced in comparison (∼0.26 au/min). This decrease in methanol dehydrogenation rate is likely due to Pt poisoning by strong binding 2,3-butanedione and methyl groups resulting from decarbonylation.

### Inelastic Neutron Scattering

The neutron vibrational spectra acquired after the adsorption of select di/ketones (acetone, 2,3-butanedione, 3,4-hexanedione, and mesityl oxide) to a Pt sponge each contained similar features, suggesting that comparable, if not identical, surface species, aside from adsorbed CO, were produced. The neutron vibrational spectra of the free vapor phase di/ketones are presented in Figure S8 for reference. The data are relatively noisy owing to the low surface coverage of the chemisorbed species and the relatively low specific surface area of the Pt sponge (∼36 m^2^/g). Furthermore, while an ideal 111 surface was assumed in the DFT calculations, the Pt sponge likely includes other crystal planes together with a variety of surface defects, possibly resulting in slight frequency shifts and broadening of significant bands. However, using the calculated INS spectra of adsorbed methyl and acyl surface species (Figures S9 and S10), some vibrational modes of these decarbonylation products could be assigned to statistically significant bands observed in the lower frequency regime of the INS spectra.

**Figure 5:**
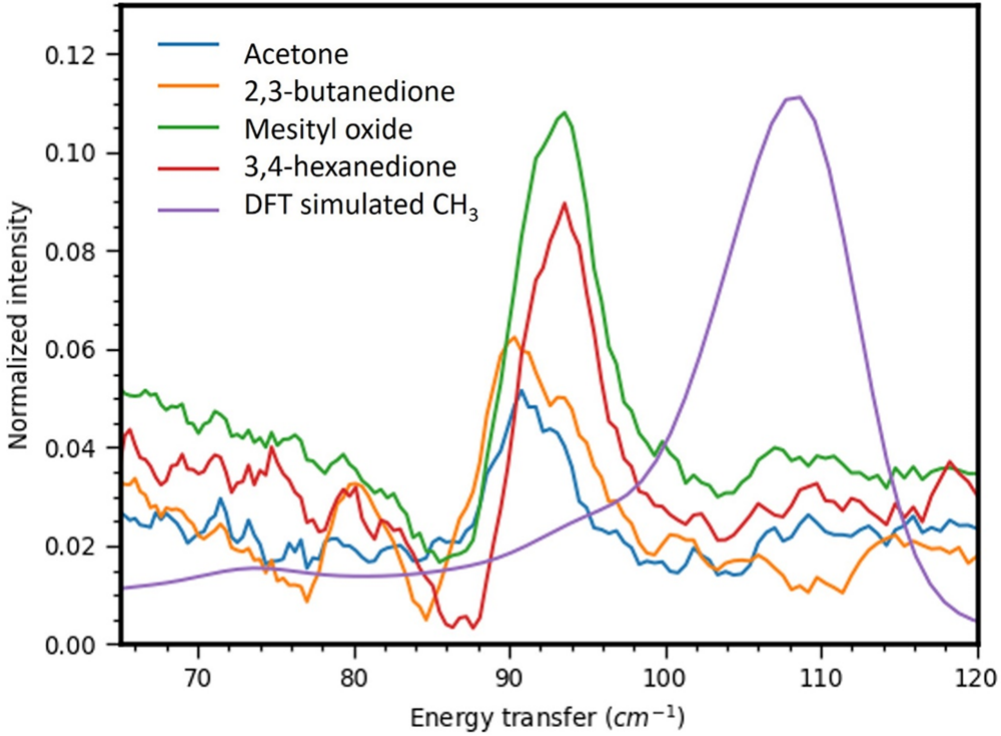
Band at 92 cm^–1^ is assigned to the Pt-CH_3_ bending mode of surface methyl groups produced from di- and ketone adsorption on a Pt sponge at 250 °C under high vacuum. The computed spectrum (purple) includes a band at 105 cm^–1^ that is representative of the Pt-CH_3_ bending mode on an ideal Pt(111) surface.

Figure 5 shows the Pt-CH_3_ bending mode around 92 cm^–1^ in each spectrum. This suggested that each di- or ketone decarbonylated to some extent at 250 °C. The Pt-CH_3_ bending mode was calculated at ∼105 cm^–1^ in DFT for CH_3_ chemisorbed on an ideal Pt(111) surface. The calculation shows that the relatively sharp band at 105 cm^–1^ is, in fact, a superposition of several bending modes that are very close in frequency (bending along different directions) on the Pt(111) surface, which accounts for the intensity of the band. Because the sharpness of the band at 92 cm^–1^ mirrors that of the computed band at 105 cm^–1^, it can be inferred that the surface CH_3_ groups are bound to structurally uniform metal sites, most likely highly coordinated terrace sites, analogous to those within the Pt(111) surface model, given their abundance throughout the Pt sponge. Due to the scarcity of more lowly coordinated edge and corner sites along with the inherent weak signal intensity of the INS spectra, the vibrational modes of any potential CH_3_ species on these sites were not exploited. Regardless, methyl groups adsorbed to highly coordinated terrace sites are of greater interest given that larger Pt particles were shown to be more active in decarbonylation as described in this manuscript as well as the methanol dehydrogenation as described elsewhere.^22^ This evidence of CH_3_ species on Pt at 250 °C corroborates the proposed consequences of carbonaceous decarbonylation fragments regarding the deactivation of supported Pt particles, as discussed during interpretation of the IR spectra.

Although Pt has a sharp phonon mode at ∼96 cm^–1^, of which the band would overlap with that of the Pt-CH_3_ bending mode, subtraction of the spectrum of bare Pt revealed residual intensity, consistent with the DFT assignment of a strong bending mode in this region. This mode is important because it forms a series of combination bands with other fundamentals. While this is complicated spectral interpretation, the presence of combination bands also helped confirm mode assignments.

**Figure 6:**
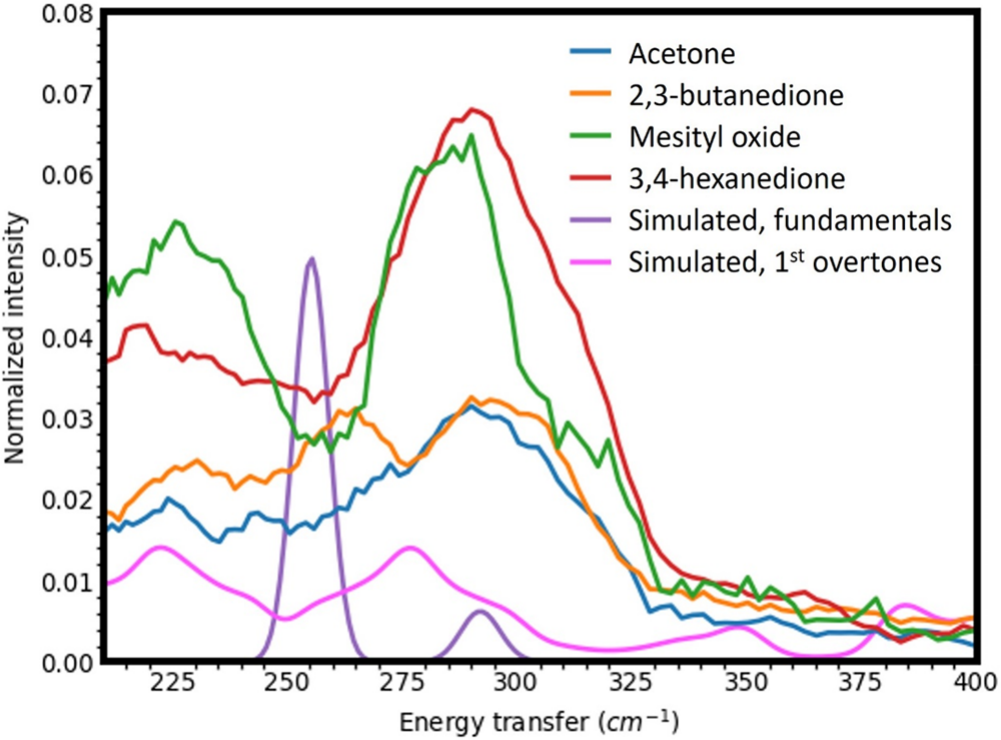
Band at 285 cm^–1^ is assigned to the Pt–C–C in-plane deformation mode of surface acyl groups produced from di- and ketone adsorption on a Pt sponge at 250 °C under high vacuum. The computed spectrum (purple) includes strong 255 cm^–1^ and weak 292 cm^–1^ bands representative of the Pt–C–C in-plane and Pt–C–O deformation modes on an ideal Pt(111) surface.

Figure 6 shows a spectral range of around 300 cm^–1^. The experimental data show a relatively strong mode at ∼285 cm^–1^. The DFT calculation showed no intensity for CH_3_–Pt(111) in this range (Figure S9), but the DFT simulation for acyl-Pt(111) showed a mode associated with the Pt–C–C in plane deformation of the acyl group at 255 cm^–1^ (Figure S10). This is one strong indication that chemisorbed acyl groups were present on the Pt sponge surface following Pt-catalyzed decarbonylation. A weaker feature around 220 cm^–1^ in the experimental spectra could be associated with an overtone of the Pt-CH_3_ bending mode, as well as a combination band of the Pt-acyl vibration at ∼100 cm^–1^ and one of the methyl torsions in the acyl group.

Attention was also given to potentially significant features with frequencies of up to 1100 cm^–1^ (Figures S11 and S12). However, bands located above 400 cm^–1^ were smaller and much broader, especially given the steady increase in the noise with increasing frequency. Some of these bands were assigned to the Pt-CH_3_ stretching (480 cm^–1^), acyl Pt–C–C out of plane bending (456 cm^–1^), H_3_C–C=O in plane deformation (564 cm^–1^), Pt-CH_3_ rocking (681 and 722 cm^–1^), and Pt-acyl rocking (903 and 967 cm^–1^) modes.

### DFT Models of Adsorbate Configurations

**Figure 7:**
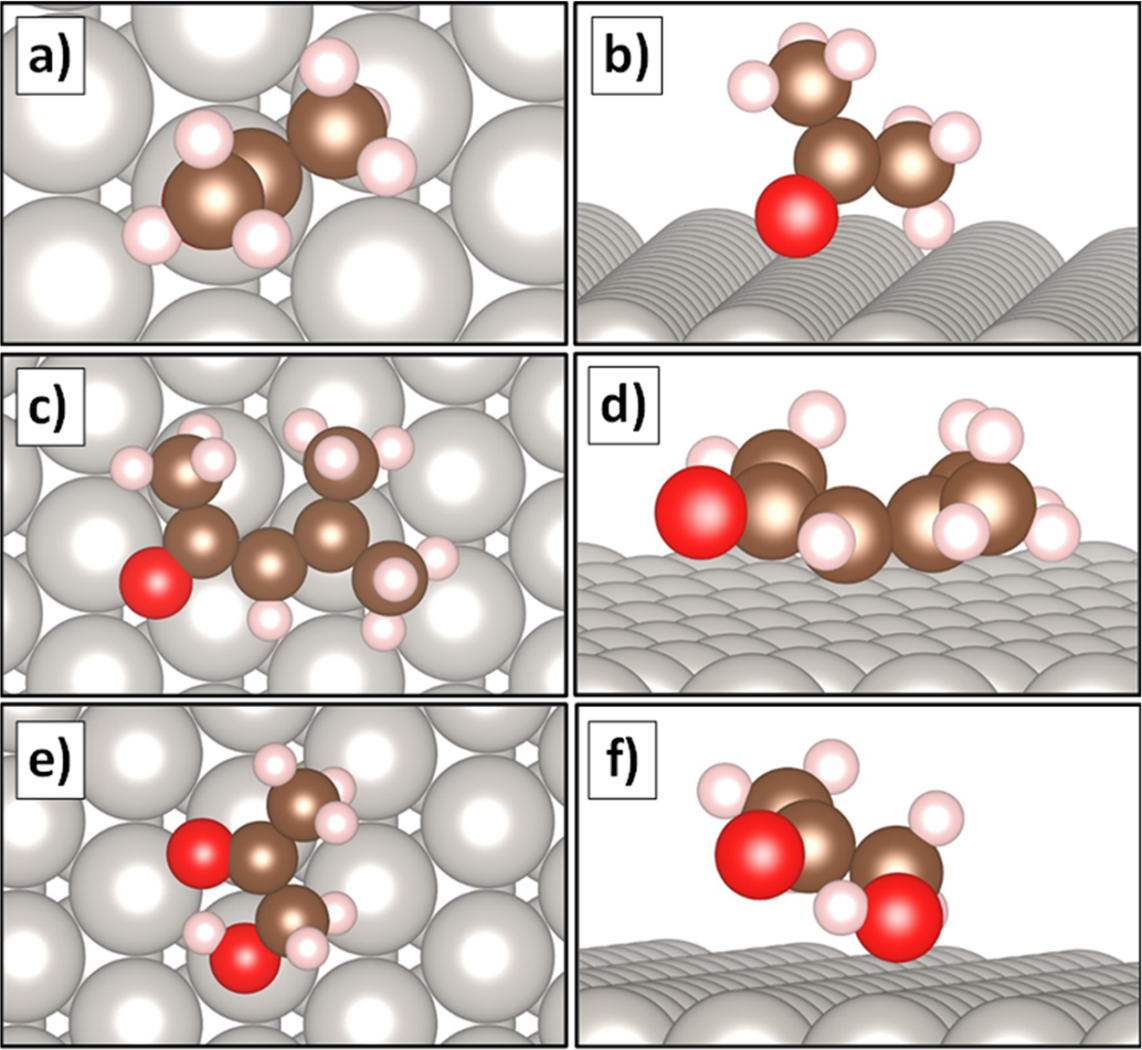
Optimized configurations of ketones adsorbed to a Pt(111) slab under vacuum. **a)** Top and **b)** side views of acetone, **c)** top and **d)** side views of mesityl oxide, and **e)** top and **f)** side views of hydroxyacetone.

Calculated binding geometries and binding energies of the various oxygenate species can provide insight into the propensity of these species to decompose. Geometries of ketones adsorbed to Pt(111), used to model binding to large Pt particles, are shown in Figure 7. Further, binding energies and Pt–O distances are provided in Table 3. Acetone adsorbed in an upright position, bound to Pt via the oxygen atom (Pt–O distance = 2.2 Å), with one of the methyl groups pointing away from the surface (Figure 7a,b). The model resembles a monodentate (η_1_) adsorbate, perhaps bound to the surface through a σ-bond. These results are in good agreement with those of previous studies that focused on modeling acetone adsorption on metallic surfaces including Pt(111).^18,55,56^ Further, the small Pt–O distance and relatively weak binding energy (see Table 3) support experimental observations that acetone will decarbonylate on large Pt particles. Mesityl oxide (Figure 7c,d), the product of acetone condensation, bound more flatly on Pt(111), suggesting more di-σ or π-bond character. This binding geometry also seems conducive to decarbonylation, with both the C=O and C=C bonds aligned parallel to the surface (Pt–O distance = 3.1 Å for this species). The methyl groups of the carbonyl and isobutyenl groups exhibited much less flexibility, seemingly unable to point away from the surface, as seen with acetone. Hydroxyacetone (Figure 7e,f) also bound to Pt with the carbonyl group parallel with the surface; however, the Pt–O distance for this group is 3.5 Å. In contrast, the adjacent alcohol group bonded directly to the surface via the oxygen atom with a Pt–O distance of 2.3 Å and a small Pt–O–C angle. Thus, the decomposition of hydroxyacetone observed experimentally could initiate along the Pt–O–C bond.

**Table 3:** Adsorption Energies and Pt–O Distances of Pt(111)-Adsorbed Di/Ketones

Structure	Adsorption Energy (eV)	Pt–O distance(s) (Å)
Acetone	–0.80	2.2
Mesityl Oxide	–1.07	3.1
Hydroxyacetone	–0.99	2.3 (OH), 3.5 (CO)
2,4-Pentanedione	–1.08	2.8, 3.3
2,3-Butanedione	–1.55	2.1, 2.1
3,4-Hexanedione	–1.10	2.1, 2.9

**Figure 8:**
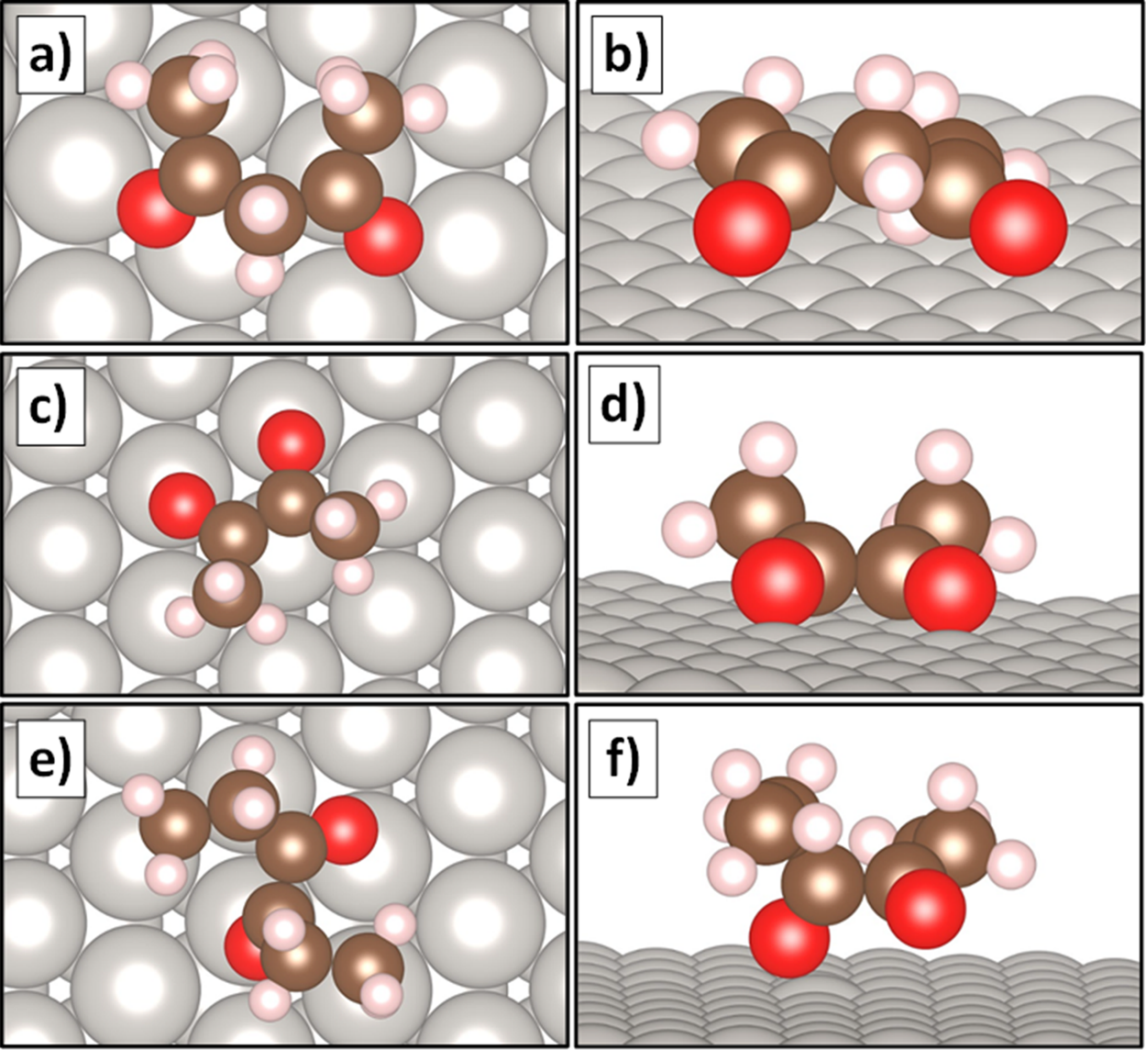
Optimized configurations of diketones adsorbed to a Pt(111) slab under vacuum. **a)** Top and **b)** side views of 2,4-pentanedione, **c)** top and **d)** side views of 2,3-butanedione, and **e)** top and **f)** side views of 3,4-hexanedione.

Diketone configurations are listed in Figure 8. Adsorbed 2,4-pentanedione (Figure 8a,b) appeared to bind nearly flat on Pt(111). In addition, the carbonyl groups point in different directions with wide O=C–C–C=O angles and Pt–O distances of 2.8 and 3.3 Å. This binding geometry seems conducive to decarbonylation, in agreement with experimental observations of large Pt particles. As for 2,3-butanedione (Figure 8c,d), the corresponding surface species also binds flat. The symmetric orientations of these diketones resemble bidentate adsorbates with a di-σ or π-bond character. The binding geometry of this species, including the small Pt–O distances of 2.1 Å, suggests that decarbonylation activity of this adsorbate should be high. This contrasts with experimental observations on large Pt particles, which showed minimal decarbonylation. This could be due to the stronger binding energy of 2,3-butanedione of −1.55 eV (−150 kJ/mol), which is notably larger than the other species investigated in this work, which all had binding energies closer to −1.0 eV (−96 kJ/mol). In contrast, the optimized configuration for adsorbed 3,4-hexanedione (Figure 8e,f) binds with the ethyl groups pointing away from the surface. In addition, the adsorbate is asymmetric, with two distinguishable carbonyls. One carbonyl group appeared to be upright with respect to the surface, potentially with a η_1_ interaction (Pt–O distance = 2.1 Å). The other carbonyl group adsorbed in a flatter orientation but may still be close enough to the surface to maintain a weak bond (Pt–O distance of 2.9 Å). The small Pt–O distances of this adsorbate support experimental observations of a high decarbonylation activity.

### Thermodynamics of Potential Reaction Paths of Surface Methyl Groups

In principle, the adsorbed CO produced by di- and ketone decarbonylation should be accompanied by the alkyl groups originally bound to the carbonyl group(s). In the case of acetone, two methyl groups should be produced per adsorbing CO species. In order to gauge the energy required to remove these species from the surface, the energies of reactions involving methyl groups on Pt(111) were calculated (Table S2). The reaction energy for acetone decarbonylation, −1.55 eV, was also calculated for direct comparison and indicates that the reaction is thermodynamically favorable on the Pt terrace sites. However, the dehydrogenation of a methyl group to form a methylene group was considered and calculated to have a reaction energy of 0.05 eV, which is slightly thermodynamically unfavorable. In addition, previous work by Viñes et al. has shown that the dehydrogenation of methyl groups on the Pt(111) surface has a relatively high activation barrier.^57^ For this reason, some alternative pathways to convert and remove the methyl groups entirely are presented in Table S2. These include associative desorption and reforming by H_2_O to form CO and H_2_. Association would result in either methane or ethane with calculated reaction energies of 0.73 and 0.48 eV, respectively. Reforming to form adsorbed CO, in theory, could occur through either an Eley–Rideal (−2.32 eV) or Langmuir Hinselwood (−1.84 eV) mechanism. While reforming appears to be a potential route for removing Pt-bound methyl groups, these calculations are only speculative given the absence of H_2_O in the model and would require further study. While solvation will likely influence the exact energies reported herein, it will not influence the overall conclusions that are presented.

Given the thermodynamic preference for the reforming of surface methyl groups by H_2_O into adsorbed CO, the reaction was attempted on 2,3-butanedione-poisoned Pt_L_/γ-Al_2_O_3_ with 1 and 10 mbar of H_2_O vapor at 150 °C (Figure S13). This would provide insight into the energy input required to facilitate methyl group chemistry. The initial adsorption of 2,3-butanedione resulted in adsorbed CO_L_, and therefore methyl groups, due to some extent of decarbonylation. During a 10-min exposure of the poisoned catalyst to 1 mbar of H_2_O, a notable reduction in the CO_L_ band occurred simultaneously with the emergence of a band at about 1570 cm^–1^. This band was associated with the ν_as_(O–C–O) mode of a formate surface species suggesting an extent of the water–gas shift took place on large Pt particles.^22^ During the subsequent increase in H_2_O vapor pressure to 10 mbar, the band at 1570 cm^–1^ continued to increase dramatically over 10 min although little changes were observed with the CO_L_ band. While this could suggest that methyl groups were reformed into adsorbed CO species that were short-lived due to water–gas shift activity, further research with other techniques would be necessary for confirmation. Although the calculated reaction energies for methyl group reforming of −1.84 or −2.32 eV point out that the process is thermodynamically favorable, the kinetic barrier may be more challenging to surpass.

## Discussion

### Decarbonylation of Di/Ketones on Small and Large Pt Particles

The size of supported metal particles in catalysts determines the distribution of surface sites, which often vary in activity to facilitate the creation or cleavage of specific chemical bonds.^58^ For decarbonylation of adsorbed di/ketones, the difference in activity and sensitivity to poisoning between small and large Pt particles is expected to be due to the configuration of the reactant on different metal sites.

For instance, di/ketones that decarbonylated readily, such as acetone and 3,4-hexanedione, appeared most stable on Pt(111) with an upright position. This aligns well with other studies that have described the interaction as a σ-bond between the carbonyl oxygen and metal site with notable repulsion between the surface and alkyl groups.^18,55,56,59^ Other di/ketones that resisted conversion and poisoned the Pt particles, such as 2,3-butanedione and mesityl oxide, maintained relatively flat configurations that resembled either π-bonds (between the C=O bond and the metal site) or di-σ bonds (distinct Pt–O and Pt–C bonds). These bonds are energetically stronger than the σ-bond of molecules in the upright configuration and result in poisoning of the Pt surface if the repulsion between the surface and alkyl groups is less significant in comparison. However, it was reported in another study that the flat-binding species acts as the precursor for acetone decarbonylation into adsorbed CO and methyl groups on Pt(111),^56^ suggesting that an ideal combination of attractive and repulsive forces may be necessary to facilitate decarbonylation. Without an adequate balance between these forces, adsorbed di/ketones remain strongly bound to the Pt surface and hinder catalytic activity. At reaction temperatures, the varying extents of repulsion when di/ketones are adsorbed to highly and lowly coordinated Pt sites may serve as a descriptor for decarbonylation activity of different metal particle sizes.

The absorbance intensity of the CO_L_ band sufficed as a gauge for comparing conversion of methanol and di/ketones on a given Pt/γ-Al_2_O_3_ catalyst. Overall, the larger metal particles of Pt_L_/γ-Al_2_O_3_ appeared more active in decarbonylation as indicated by the larger CO_L_ bands observed during di/ketone adsorption (Figures 3 and 4). Considering the higher abundance of highly coordinated surface sites in the larger particles, these observations agree with the role of repulsive interactions established by the studies of the model systems mentioned above. Furthermore, the subsequent adsorption of methanol on these larger Pt particles added to the intensity of this band to closer match that of its clean counterpart indicating less pronounced poisoning by the di/ketones or their fragments (Table 2). This is strong evidence that smaller Pt particles are more prone to poisoning in comparison to larger ones. However, given the diversity of di/ketones studied on Pt/γ-Al_2_O_3_ catalysts, there are several chemical species that need to be discussed: alkyl and acyl groups (decarbonylation fragments) and molecular di/ketones.

### Poisoning by Alkyl Groups and Derivatives

The deactivation of supported metal catalysts by strong binding of kinetically stable surface methyl groups has been observed in a few cases involving other metals and reactions. Albers et al. reported that strongly adsorbed methyl groups on a Pd catalyst originated from side reactions in various industrial chemical processes including the hydrogenation of functionalized aromatics.^60^ Not only can these methyl groups occupy metal sites and hinder adsorption of reactants but large coverages may alter the surface polarity and thus any remaining ability of the metal to catalyze the intended reaction. Considering that other APR studies have claimed catalyst deactivation by coke formation,^11,61,62^ these methyl and acyl groups may potentially engage in subsequent dehydrogenation and aromatization reactions to produce heavy coke deposits on metal particles. Since the formation of surface methyl groups (and other alkyl groups) occurs concomitantly with di/ketone decarbonylation, the effects of these surface species herein cannot be ignored.

Ideally, this would include studies of spent catalysts from APR reactions, but detecting and quantifying methyl groups on supported metal catalysts come with several challenges. Importantly, it is known that significant amounts of carbonaceous deposits form on the γ-Al_2_O_3_ support under typical conditions.^63,64^ These species can block access to supported metal sites when they happen to be at the perimeter of the metal particles, but due to their abundance, it would be very difficult to identify the more potent poisons that may reside on the metal particles considering that metal loading on Pt/γ-Al_2_O_3_ catalysts is typically only 1–5 wt %.^54^ While the alkyl groups were not directly detected by IR spectroscopy, the experiments in the present study suggested that alkyl groups make up a notable coverage on Pt when an adsorbed di/ketone readily decarbonylates. For instance, preadsorbed acetone led to a 65% decrease in the CO_L_ band integral on Pt_L_/γ-Al_2_O_3_ during subsequent methanol dehydrogenation. Given the 2:1 stoichiometric formation of CH_3_:CO surface species during acetone decarbonylation, it is likely that this severe extent of poisoning is due to a nearly 65% coverage by surface methyl groups. The INS spectra (Figures 5, 6, S11, and S12) not only presented evidence for the persistent presence of these species under high vacuum but also showed that they are stable at 250 °C, a temperature at which APR is commonly operated.

Given the thermodynamic stability of methyl groups and other alkyl species,^65^ there are limited options to remove them via a thermodynamically favorable reaction path. The reaction energies calculated herein (Table S2) show that the reforming of methyl groups into adsorbed CO is very favorable, while association of methyl groups into CH_4_ or C_2_H_6_ has little energetic consequences. However, the calculations do not account for the presence of bulk H_2_O used under actual APR conditions or the energy required to surpass the activation barrier. Further experiments would be needed to determine at which temperatures these reactions occur and to calculate the respective activation energies. If the energetic cost is high, then a separate catalyst regeneration procedure, such as oxidation with O_2_ dissolved in H_2_O, may be necessary.

Acyl groups were also observed on the Pt sponge at temperatures as high as 250 °C, as suggested by the 285 cm^–1^ band assigned to the respective Pt–C–C in-plane deformation mode. This includes acetyl groups afforded by acetone and 2,3-butanedione partial decarbonylation as well as propionyl groups resulting from 3,4-hexanedione partial decarbonylation. These acyl species have been proposed to be essential intermediates during aldehyde decarbonylation on Pt(111).^66^ Similar phenomena are suggested to occur on a Pt sponge replete with highly coordinated sites during the conversion of di/ketones. Nonetheless, these acyl surface species are expected to further decompose into adsorbed CO and methyl groups.

### Poisoning by Molecular Di/Ketones

Given the lower decarbonylation activity of small Pt particles, adsorbed di/ketones are likely to retain their molecular structure and resist conversion when adsorbed to lowly coordinated Pt sites. In addition, diketones such as 2,3-butanedione and mesityl oxide still acted as strong poisons for large Pt particles. As mentioned earlier, the steric repulsion between alkyl groups and Pt terraces may drive the decarbonylation of these species on larger Pt particles. Yet, this repulsion becomes a less significant factor when adsorbed to more exposed metal sites.^18^ Without this essential driving force, lowly coordinated sites are more vulnerable to poisoning by adsorbed di/ketones.

Certain di/ketones in this study did not decarbonylate as readily as acetone at temperatures up to 250 °C yet severely hindered methanol dehydrogenation. 2,3-Butanedione was the strongest poison for large Pt particles, decreasing the CO_L_ band integral by 75%. It also exhibited the highest adsorption energy, −1.55 eV, of all of the molecular di/ketones adsorbed to Pt(111) and a relatively flat, symmetric orientation in which both carbonyl groups interact with the metal surface (Figure 8c,d). In contrast, 3,4-hexanedione, the larger α-diketone, only had an adsorption energy of −1.10 eV, bonded upright with monodenticity on Pt(111) and decarbonylated readily on Pt/γ-Al_2_O_3_ catalysts. It is suggested that the more bulky ethyl groups of 3,4-hexanedione prevent the adsorbed species from maintaining a stable flat orientation characteristic of di-σ bonds. For 2,3-butanedione, the DFT calculations predicted an adsorbed species with two di-σ bonds (or a π bond) and smaller methyl groups, which is sufficiently stable to poison large Pt particles.

Hydroxyacetone also bonded flat on the Pt(111) surface (Figure 7e,f) suggesting that the OH group stabilizes a surface species with more di-σ bond character compared to acetone. Yet, hydroxyacetone decarbonylated readily on large Pt particles (Figure 4d) with a measured poisoning extent of only 39%, but there was negligible subsequent dehydrogenation of methanol, suggesting surface coverage was dominated by hydroxyacetone-derived species. The observed pinning extent suggests that hydroxyacetone decarbonylated to near completion given that the result should be 1:2 CH_3_:CO (with some residual surface hydrogen) due to simultaneous dehydrogenation of the OH group. Just as the flat orientation is the decarbonylation precursor for adsorbed acetone, the same configuration of adsorbed hydroxyacetone with an added interaction between the Pt and OH group appears to be necessary for decarbonylation. The reaction path is in agreement with results reported by McManus et al., who showed that the formation of α-oxo-η^2^ intermediates was essential for the reforming of C_3_ aldoses on Pd.^15^ While the study focused on the conversion of aldehydes, hydroxyacetone in this study still appeared to decarbonylate at temperatures as low as 50 °C, supporting this mechanism.

The few studies focusing on the adsorption of conjugated species onto metal surfaces have generally taken theoretical approaches. For instance, Loffreda studied the adsorption of several different conjugated species on a Pt(110) surface and revealed that conjugated ketones bind more strongly to metal surfaces than other conjugated alkenes with other functional groups (i.e., carboxy, nitro, imino) due to destabilization of the highest occupied molecular orbital (HOMO) of the molecule.^67^ In another study, the same author reported that the trans configurations of these species generally bind flat on metallic surfaces with up to 4-fold hapticity on Pt.^68^ The configurations reported herein for adsorbed mesityl oxide on Pt(111) are consistent with these studies (Figure 7c,d), and the combined binding of both the C=O and C=C groups to the metal surface may explain the enhanced adsorption energy, −1.07 eV, compared to that of adsorbed acetone, −0.80 eV. However, ketone-alkene conjugation, and thus HOMO stabilization, was also reported to reduce the repulsion between the metal surface and the nonbonding alkyl components of the adsorbed species.^67^ This in part explains why mesityl oxide did not poison large Pt particles as much as 2,3-butanedione.

It is known that aldol self-condensation of acetone into mesityl oxide occurs readily on Lewis acidic materials, including γ-Al_2_O_3_.^49,50,53,69^ It was also previously reported that several of the di/ketones employed in this study undergo some extent of enolization and aldol condensation to form bulky conjugated products when adsorbed to γ-Al_2_O_3_ at temperatures as low as 250 °C.^54^ This is important to consider when discussing the susceptibility of interfacial sites. Many of the metal sites on the small Pt particles of Pt_S_/γ-Al_2_O_3_ are within close proximity to the γ-Al_2_O_3_ support on which these condensation products are formed and may continue to reside. Thus, it is to be expected that molecules adsorbed on the support, including but not limited to conjugated aldol self-condensation products, can reduce the methanol dehydrogenation activity of interfacial sites by both steric hindrance and the possibility of direct binding of these multifunctional surface species to the respective metal sites. The enlargement of Pt particles should alleviate these effects, given the greater abundance of coordinated metallic sites that are sufficiently distant from the Lewis acidic support.

### Possibilities for Improving APR Catalyst and Process Robustness

The formation of alkyl groups and dehydrogenation products on supported Pt particles is inevitable, given the extensive array of APR reactants. Thus, their impact on the APR process must be managed by proper catalyst and reactor design. One option could be to cofeed small amounts of dissolved O_2_ or apply mild oxidation as a periodic regeneration. Interestingly, the presence of H_2_O reduces the activation barrier for oxidation reactions for some surface species including adsorbed CO.^70,71^ However, the oxidation of surface species would likely be nonselective, resulting in a reduced H_2_ yield by oxidation of surface hydrogen. Therefore, adjustments should be made directly to the catalyst design to achieve a material that can passively remove alkyl groups with minimal or positive effects on the APR efficiency and H_2_ yields.

Ni for instance has demonstrated high APR activity with innate selectivity to light alkanes.^72^ Trace additions of the metal to Pt particles may prove useful for removing alkyl groups through the association with surface hydride species. However, in the case of methyl groups, the resulting methane is an undesired product given it is a potent greenhouse gas and requires substantial energy to be reformed to increase the H_2_ yield. On the other hand, oxyphilic promoters may prove capable of oxidizing or reforming surface alkyl groups without disrupting the intended conversion of adsorbed CO. A study by Michalak et al. demonstrated enhanced CO oxidation activity over a supported PtSn bimetallic catalyst with segregated Pt and Sn domains.^73^ The Sn domains acted as oxygen reservoirs and reduced the activation energy at the Pt–Sn interface (compared to that of the CO oxidation on a monometallic Pt catalyst). In the context of APR, a multicomponent catalyst that consists of local oxygen reservoirs that may selectively oxidize alkyl groups into adsorbed CO or light alcohols may prove to be more practical. This would require further study to ensure that adsorbed CO or other crucial APR intermediates are not unintentionally converted.

The results herein have also suggested that mesityl oxide and potentially other conjugated species formed from aldol condensation on the γ-Al_2_O_3_ support may act as strong poisons for both metallic and interfacial Pt sites. Koichumanova et al. utilized IR spectroscopy to study the APR of hydroxyacetone with Pt/γ-Al_2_O_3_ and Pt/ZrO_2_ catalysts and observed no formation of adsorbed CO.^74^ Yet, they addressed the formation of conjugated products from the aldol condensation of hydroxyacetone, which may have been responsible for the lack of conversion. However, Justicia et al. reported successful APR of hydroxyacetone into mixtures of H_2_, CO_2_, and CH_4_ using a carbon black-supported Pt catalyst with no mention of conjugated species.^75^ Because the condensation of di/ketones can be facilitated by the Lewis acid sites of γ-Al_2_O_3_,^54^ the employment of a more inert support should circumvent this side reaction and reduce the extent of Pt poisoning.

## Conclusions

Poisoning of Pt/γ-Al_2_O_3_ by strongly binding ketones, diketones, and their fragments is investigated to elucidate the limited efficacy of aqueous phase reforming of these molecules. Using IR spectroscopy, it is shown that the adsorption of various di/ketones and their products can severely poison Pt particles of different sizes as indicated by the hindrance of subsequent methanol dehydrogenation, which readily results in a high coverage of adsorbed carbon monoxide on a clean surface. Additional results from density functional theory and inelastic neutron scattering indicate that Pt/γ-Al_2_O_3_ catalysts are poisoned by a combination of molecular di- and ketones, alkyl and acyl groups resulting from di- and ketone decarbonylation, and conjugated ketones. Small Pt particles are highly vulnerable to poisoning by molecular diketones due to lesser decarbonylation activity, while their relatively abundant interfacial sites are blocked by conjugated species bound to γ-Al_2_O_3_, which are formed by aldol self-condensation of di/ketones. Although larger Pt particles appear more active in di/ketone decarbonylation, modeling of di/ketones adsorbed to Pt(111) suggests that some di/ketones with a thermodynamically preferred flat configuration (i.e., 2,3-butanedione, mesityl oxide) can still resist decarbonylation potentially due to greater adsorptive hapticity, insufficient repulsion between the metal surface and alkyl groups, and insufficient intramolecular repulsion between polar carbonyl and apolar alkyl groups. However, inelastic neutron scattering spectra suggest the presence of alkyl and acyl groups from di/ketone decarbonylation (e.g., methyl or acyl groups from acetone) on a Pt sponge that remain adsorbed at temperatures as high as 250 °C. Calculated reaction energies further imply that the removal of surface methyl groups, under high vacuum, by associative desorption or partial oxidation is energetically unfavored, and the removal of such species may require catalyst regeneration or the design of improved catalysts. The findings and perspectives herein provide potential directions for these improvements of aqueous phase reforming.

## Supporting Information Available

Unit cells for DFT simulations, full range (1000–4000 cm^–1^) IR spectra of di/ketones and methanol adsorbed on Pt catalysts, semiquantitative analysis of CO_L_ bands, additional INS spectra, calculated methyl group reaction energies on Pt(111) (PDF)
