# Supplementary material for: Poisoning of Pt/γ-Al2O3 Aqueous Phase Reforming Catalysts by Ketone and Diketone-Derived Surface Species
Source: ACS Catal. 2024 Jan 16;14(3):1480–93. doi: 10.1021/acscatal.3c04774 (PMC10845116; doi:10.1021/acscatal.3c04774)
Supplement: Supplementary file 1 — cs3c04774_si_001.pdf [file cs3c04774_si_001.pdf]

Supporting Information for:

Poisoning of Pt/γ-Al_2O_3 aqueous phase reforming catalysts by ketone and diketone-derived surface species

Bryan J. Hare[1], Ricardo A. Garcia Carcamo[2], Luke L. Daemen[3], Yongqiang Cheng[3], Rachel B. Getman[2], Carsten Sievers[1]*

[1]School of Chemical & Biomolecular Engineering, Georgia Institute of Technology, Atlanta, GA 30332, United States

[2]Department of Chemical and Biomolecular Engineering, Clemson University, Clemson, SC 29634, United States

[3]Spallation Neutron Source, Oak Ridge National Laboratory, Oak Ridge, TN 37830, United States

*Corresponding author: carsten.sievers@chbe.gatech.edu

Two types of simulation supercells for DFT calculations were used depending on the type of calculation: a larger supercell (Figure S1) that can accommodate the larger adsorbates used in the result section of the main text for binding and reaction energies and a smaller supercell (Figure S2) to accommodate CH_3*, a relatively small adsorbate for the prediction of the neutron vibrational spectrum. The first and larger supercell is required to test the larger set of adsorbates and the second and smaller supercell is required to make the neutron vibrational spectrum estimation computationally tractable.

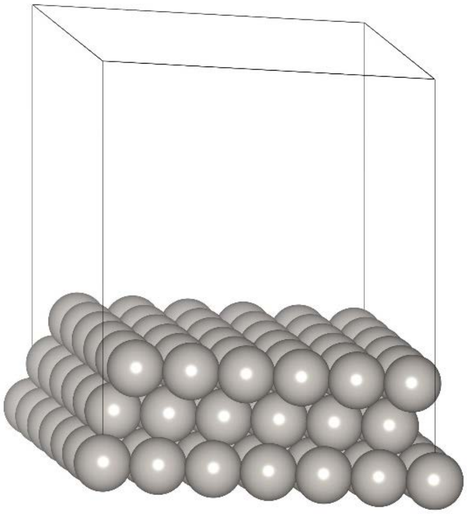

Figure S1. Larger DFT simulation supercell used for binding and reaction energies.

To test the impact of adding a fourth atomic layer on the unit cell for the data used in Table 3 in the main text; the binding energy for acetone and 3,4 hexanedione was calculated and compared with the three-layer model. In Table S1 it is shown that for medium size adsorbate like acetone the difference in the binding energy is below 0.03eV and for 3,4 hexanedione (the largest adsorbate) the difference is below 0.1 eV. This difference is with expected margins for DFT methods.

Table S1. Testing for the impact of adding a fourth atomic layer on binding energies.

Adsorbate	Binding Energy (eV)	
	3 Layers	4 Layers
Acetone	-0.80	-0.78
3,4 hexanedione	-1.09	-1.01

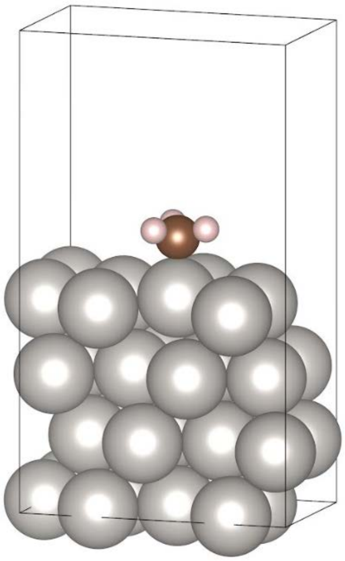

Figure S2. Smaller DFT simulation supercell used for the neutron vibrational spectrum.

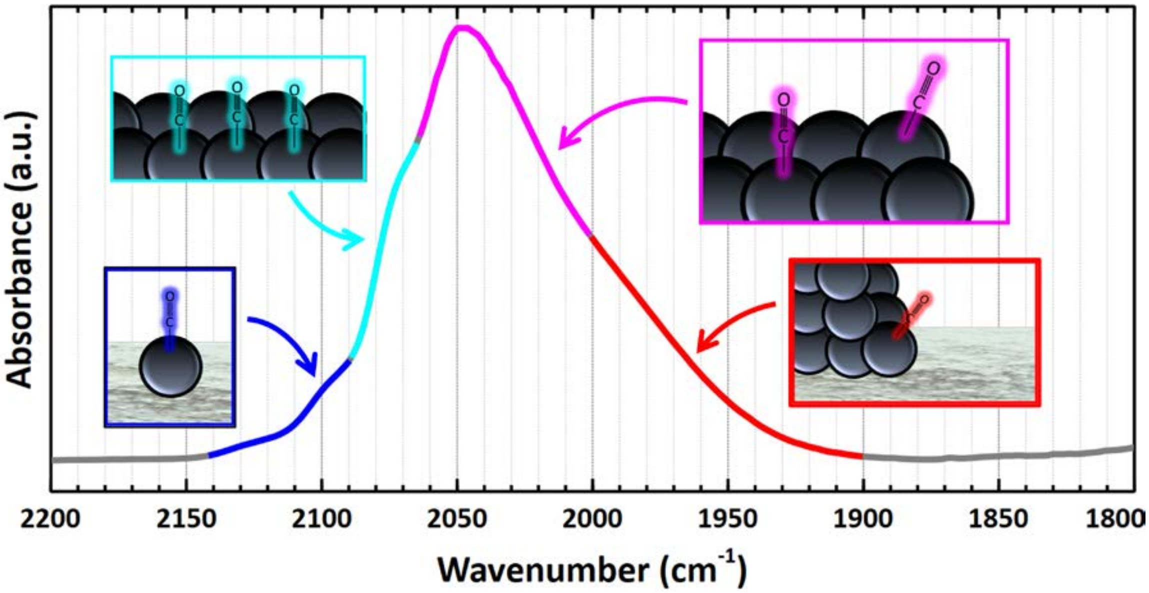

Figure S3. Infrared band assignments for CO adsorbed to Pt/γ-Al_2O_3 under high vacuum.

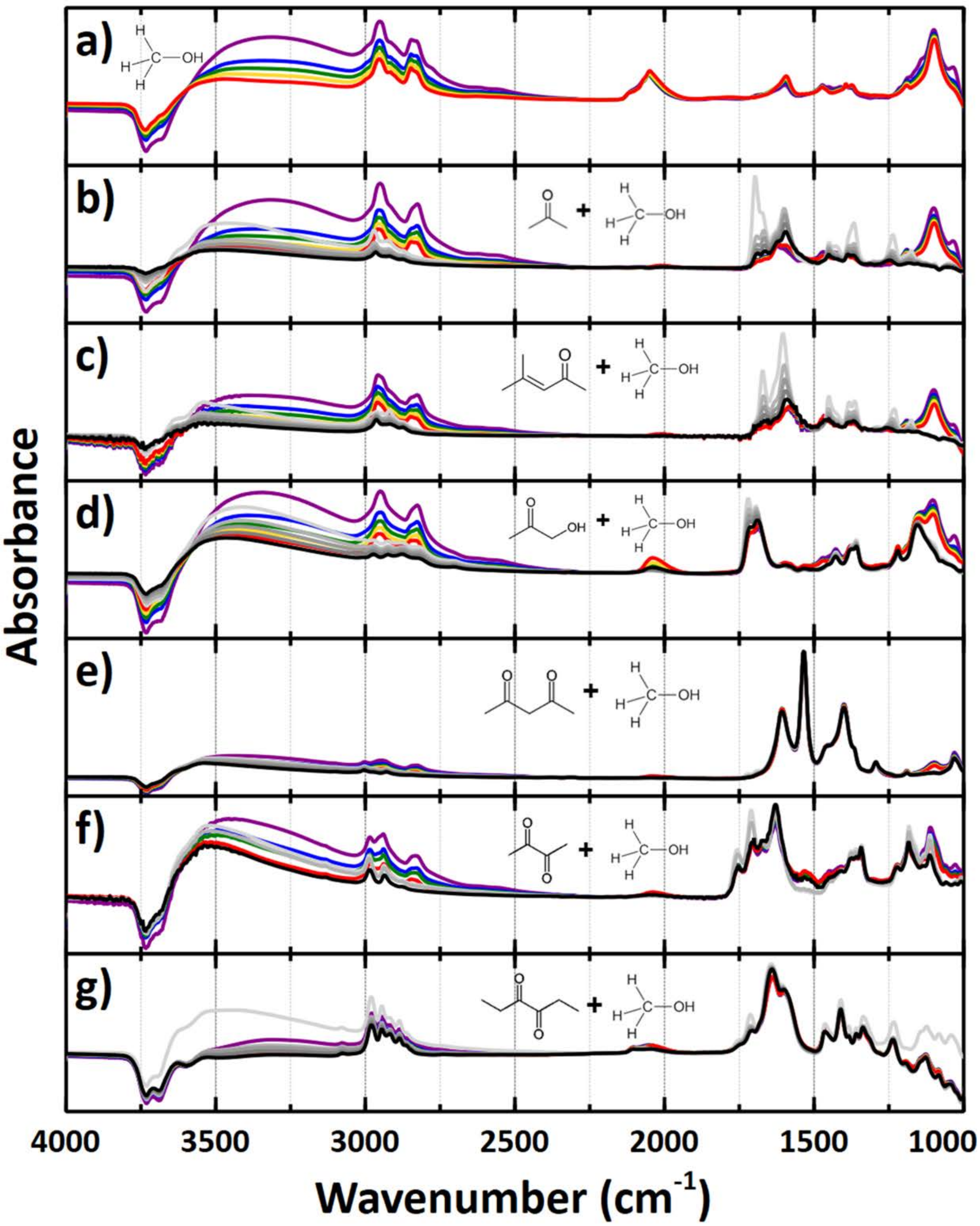

Figure S4. Full IR spectra during TPD experiments with $Pt_S/\gamma\text{-}Al_2O_3$ (~1.1 nm Pt particles) up to 250 °C. **a)** Methanol TPD on clean $Pt_L/\gamma\text{-}Al_2O_3$. Methanol TPDs following poison TPDs in which the poison is **b)** acetone, **c)** mesityl oxide, **d)** hydroxyacetone, **e)** 2,4 pentanedione, **f)** 2,3 butanedione, and **g)** 3,4 hexanedione.

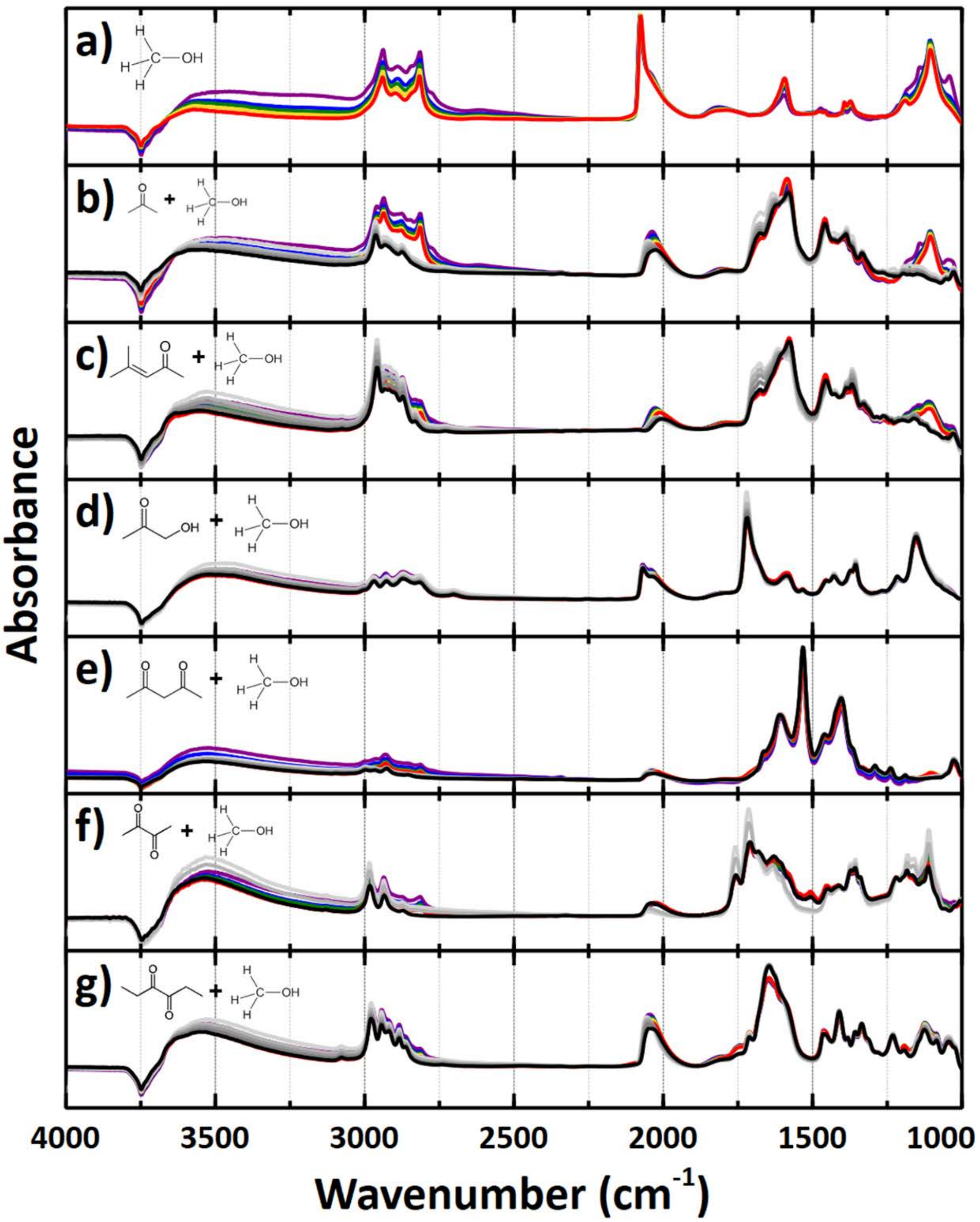

Figure S5. Full IR spectra during TPD experiments with Pt_L/γ-Al_2O_3 (~4.6 nm Pt particles) up to 250 °C. **a)** Methanol TPD on clean Pt_L/γ-Al_2O_3. Methanol TPDs following poison TPDs in which the poison is **b)** acetone, **c)** mesityl oxide, **d)** hydroxyacetone, **e)** 2,4 pentanedione, **f)** 2,3 butanedione, and **g)** 3,4 hexanedione.

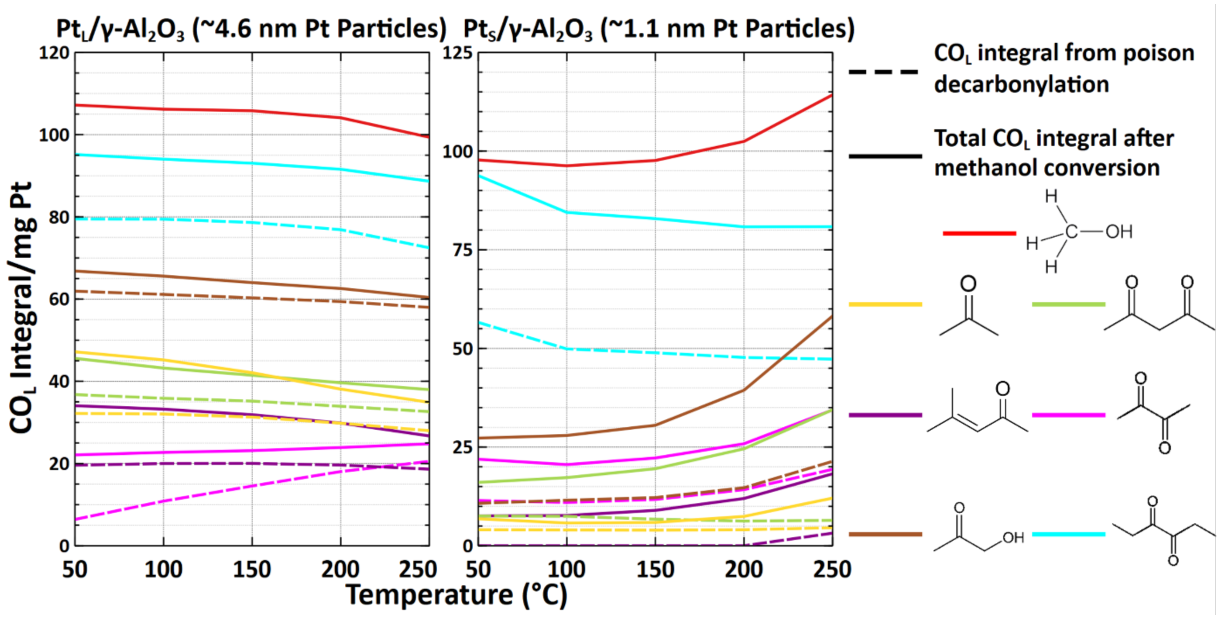

Figure S6. Integrals of the ν(C≡O) IR band during poison adsorption (dashed lines) and subsequent methanol adsorption (solid lines) from 50 to 250 °C on both small and large Pt particles (left and right, respectively).

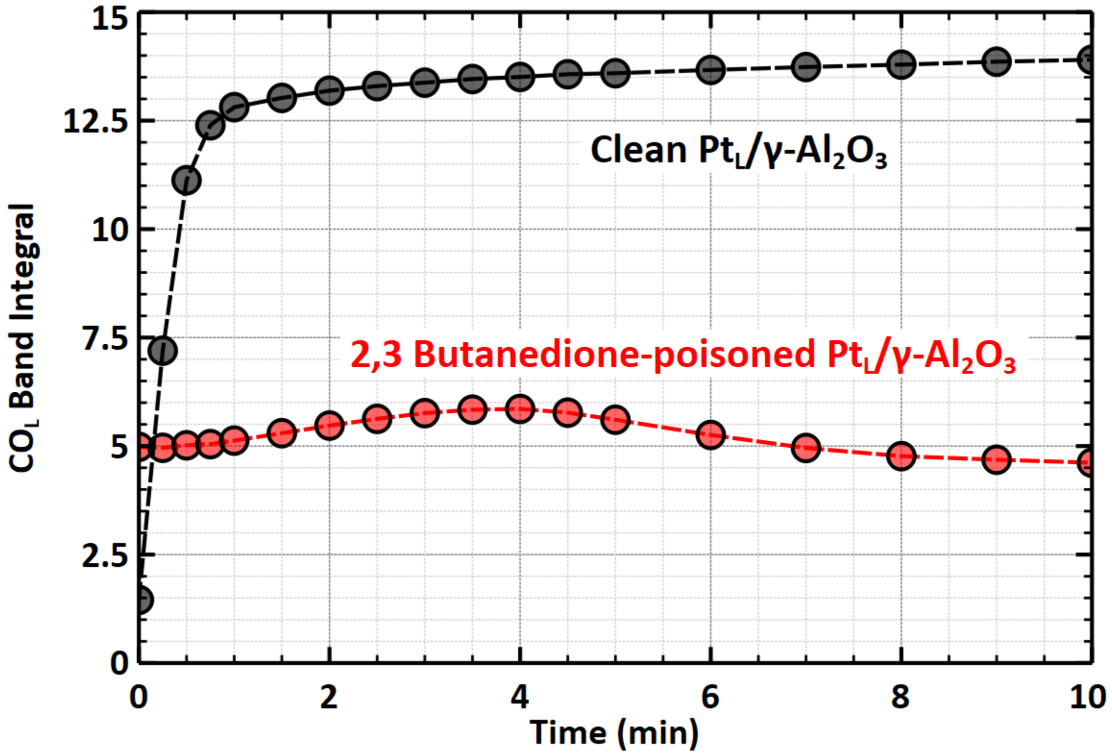

Figure S7. CO band integrals during methanol dehydrogenation on clean and 2,3 butanedione-poisoned Pt$_L$/γ-Al$_2$O$_3$ at 150 °C.

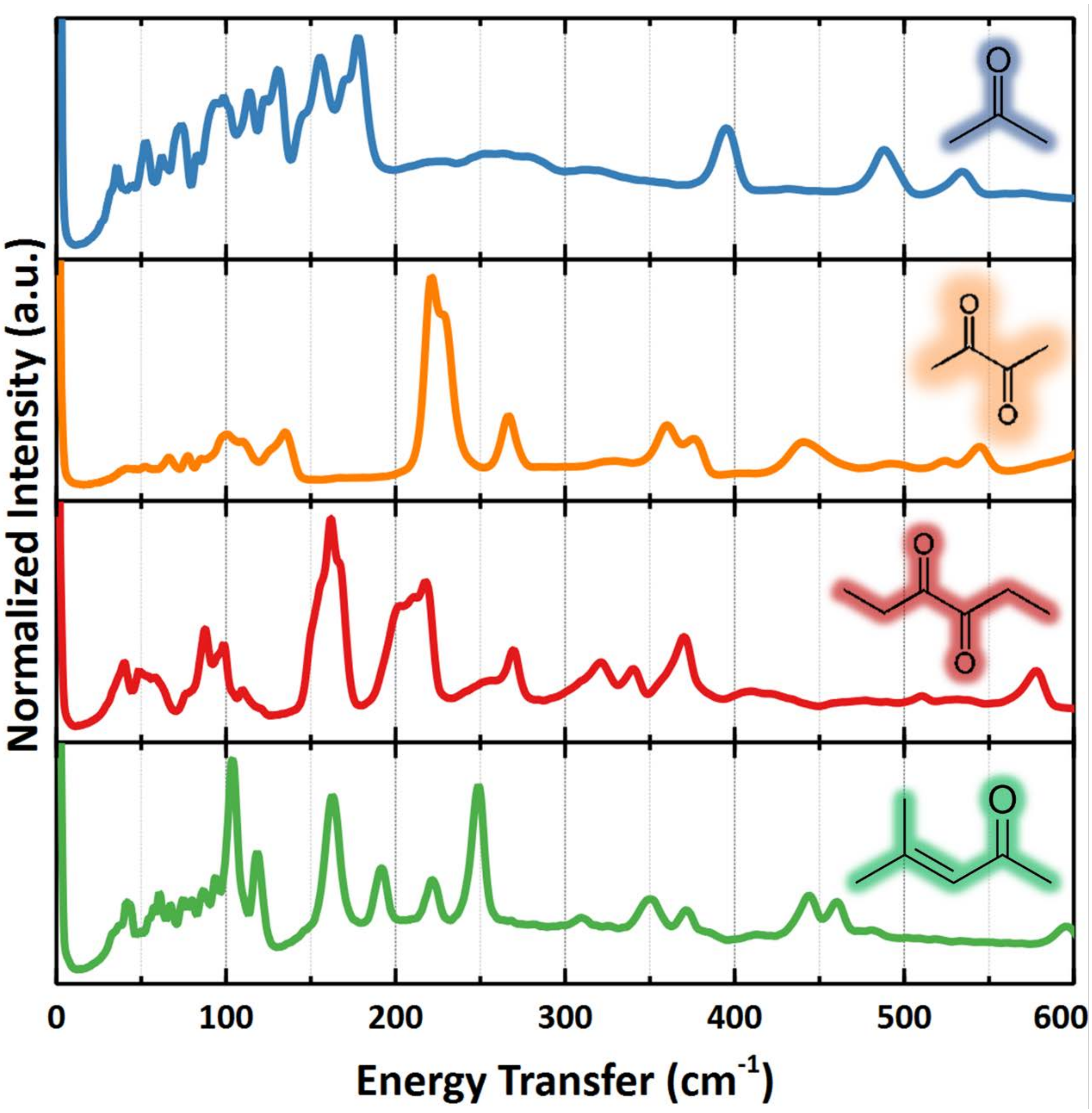

Figure S8. Reference inelastic neutron scattering spectra for some free di/ketone reagents.

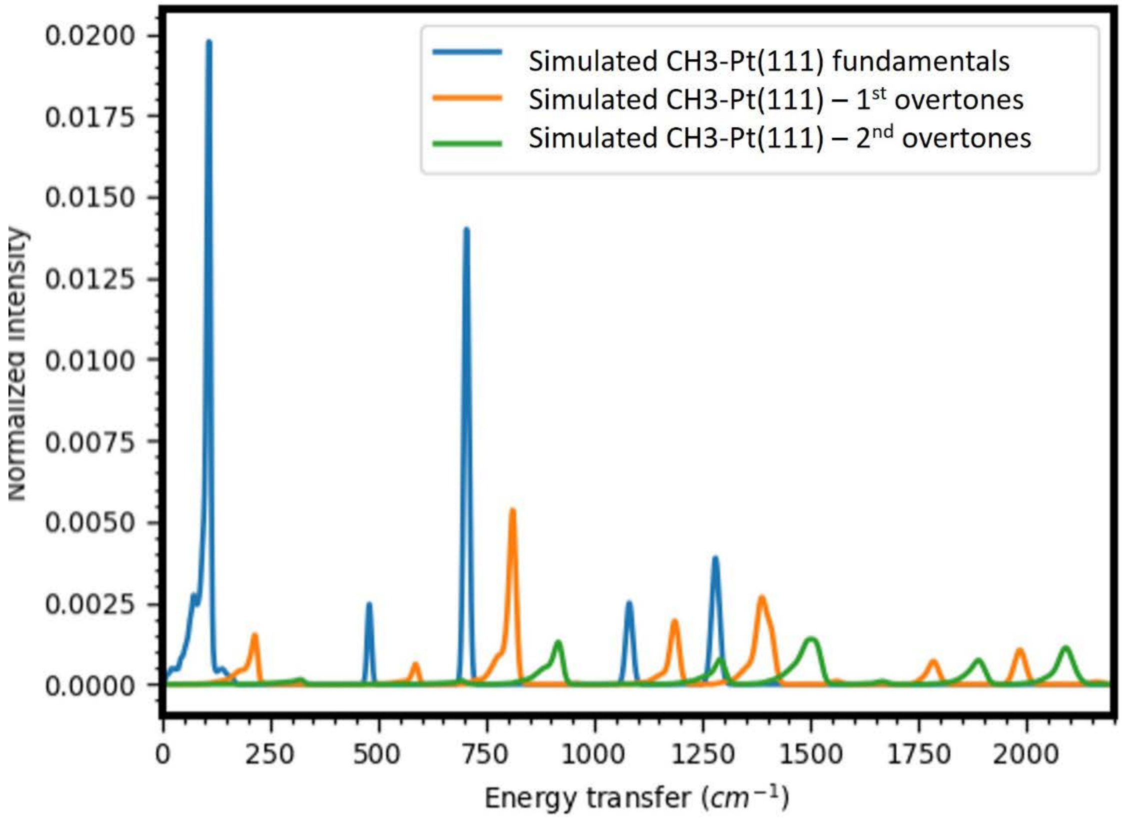

Figure S9. Calculated neutron vibrational spectrum of a single methyl group chemisorbed on a Pt 111 surface. Fundamentals are shown in blue. Modes associated with first overtones are shown in orange, and modes pertaining to second overtones appear in green. The experimental spectrum is the sum of these contributions.

Figure S9 shows the calculated vibrational spectrum for a methyl group attach to a Pt atom on the 111 facet of a Pt slab. The chemisorbed methyl group has a strong (large amplitude of motion) bending mode around 106 cm^{-1}. This strong mode forms combination bands with the Pt-CH_3 stretch, rocking, umbrella, and scissoring modes, which complicates the analysis. In **Figure S9**, blue lines are the fundamentals (0->1 transitions). At ~480 cm^{-1} is the Pt-CH_3 stretch; 730cm^{-1} is the CH_3 rocking; 1105 cm^{-1} is the umbrella mode; 1313 cm^{-1} is the CH_3 scissor. Orange curves show the first overtones and combination bands: 218 = 2 x 106; 586 = 485 + 106; 833 = 730 + 106; 1214 = 1105 + 106; 1433 = 1313 + 106. The green curve shows intensity involving second overtones such as 939 = 723 + 2 x 106; 1531 = 1313 + 2 x 106. The presence of strong overtones and combination bands is somewhat surprising and accounts for some of the complexity of the experimental spectrum.

The spectral signatures for CH_3 are observable in the experimental data. However, a few modes are observed in the experimental spectra that are not associated with a methyl group suggesting a minute presence of acyl groups that may also result from di/ketone decarbonylation. A VASP calculation with an acyl group attached to a Pt atom reproduced several spectral features observed in the experimental data. For example, the strong band at ~290cm^{-1} in the VISION data is not associated with chemisorbed methyl (no intensity in this range in the VASP calculation). But there is a strong fundamental mode calculated at ~255 cm^{-1} which is the Pt-(C=O)-CH_3 bending mode **(Figure S10)**. Intensity at ~584 cm^{-1} is calculated at ~564 cm^{-1} by VASP and corresponds to the in-plane O=C-CH_3 bending of the acyl group. Associated CH_3 rocking modes (in plane and out of plane) are calculated at 903 and 967 cm^{-1}. The umbrella mode for -CH_3 on acyl is calculated around 1306 cm^{-1}; scissors at 1386 and 1393 cm^{-1} (compared to 1105 and 1313 cm^{-1} for Pt-CH_3, respectively).

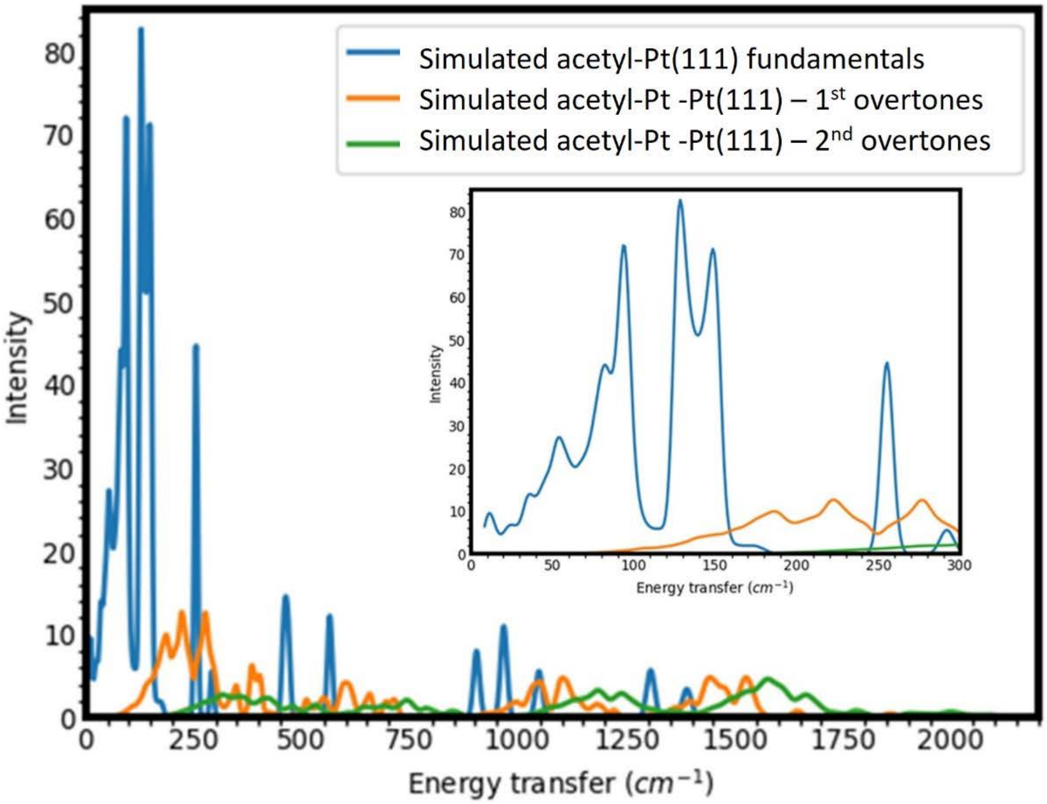

Figure S10. Calculated neutron vibrational spectrum of a single acyl group chemisorbed on a Pt 111 surface. Fundamentals are shown in blue. Modes associated with first overtones are shown in orange, and modes pertaining to second overtones appear in green. The experimental spectrum is the sum of these contributions. The inset shows the low frequency part of the spectrum. The band below 100 cm^{-1} involves modes associated with displacement of the entire acyl group, such as librations. The two strong peaks at 129 and 149 cm^{-1} are methyl torsions, while the fundamental at 255 cm^{-1} is a Pt-C bending mode accompanied by an in-plane deformation of the Pt-C-CH_3 angle.

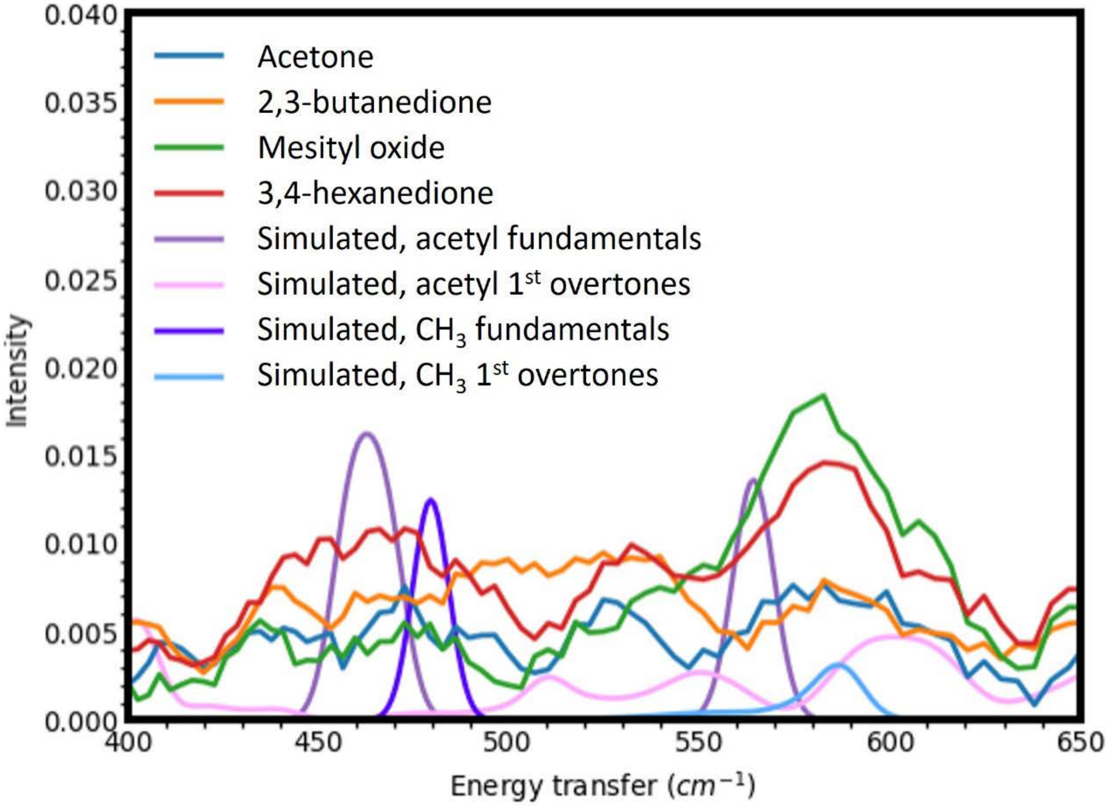

Figure S11. The 400-650 cm^{-1} spectral range. The purple curve shows two acyl group fundamentals calculated by VASP with the acyl-Pt(111) model at 456 cm^{-1} (out-of-plane Pt-C deformation) and 564 cm^{-1} (Pt stretch + O-C-methyl in-plane deformation). The associated 1st overtones are shown in pink. The dark blue curve shows a VASP CH3-Pt(111) fundamental at 480 cm^{-1} (Pt-CH_3 stretch) showing a fundamental around 480 cm^{-1} (Pt-CH_3 stretch). The light blue curve shows the corresponding 1st overtones as a combination band around 585 cm^{-1}.

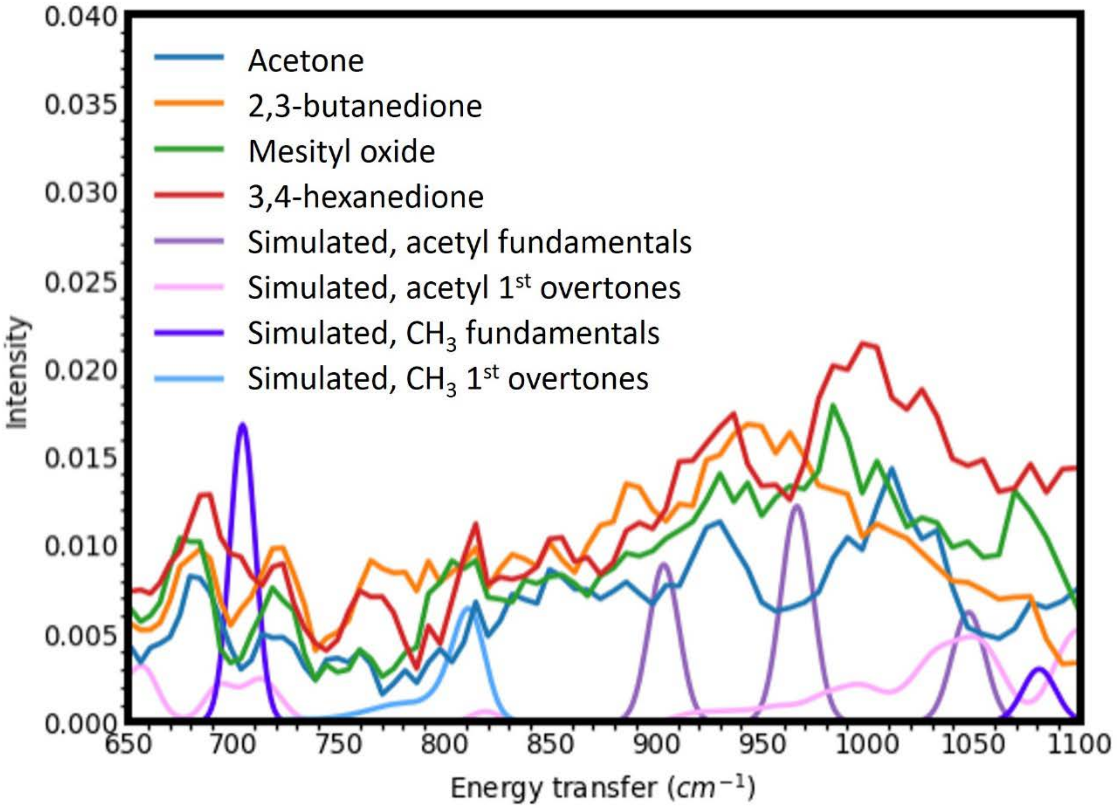

Figure S12. The 650-1100 cm^{-1} spectral range. This range shows the CH_3 rocking modes in the experimental data and calculated with the CH_3-Pt(111) (dark blue = fundamentals; light blue = 1st overtones) and acyl-Pt(111) models (purple = fundamentals; pink = 1st overtones).

Table S2. Reaction energies for processes on Pt(111) that produce and consume methyl groups under APR conditions.

Reaction		Reaction energy (eV)
**Acetone Decarbonylation**	$(CH_3)_2CO^* + 2^* \rightarrow 2CH_3^* + CO^*$	-1.55
**Methyl Dehydrogenation**	$CH_3^* + ^* \rightarrow CH_2^* + H^*$	0.05
**Methyl-Hydride Association**	$CH_3^* + H^* \rightarrow 2^* + CH_4(g)$	0.73
**Methyl-Methyl Association**	$2CH_3^* \rightarrow ^* + C_2H_6^*$	0.48
**Methyl Reforming (Eley-Rideal)**	$CH_3^* + H_2O + 5^* \rightarrow CO^* + 5H^*$	-2.32
**Methyl Reforming (Langmuir-Hinshelwood)**	$CH_3^* + H_2O^* + 4^* \rightarrow CO^* + 5H^*$	-1.84

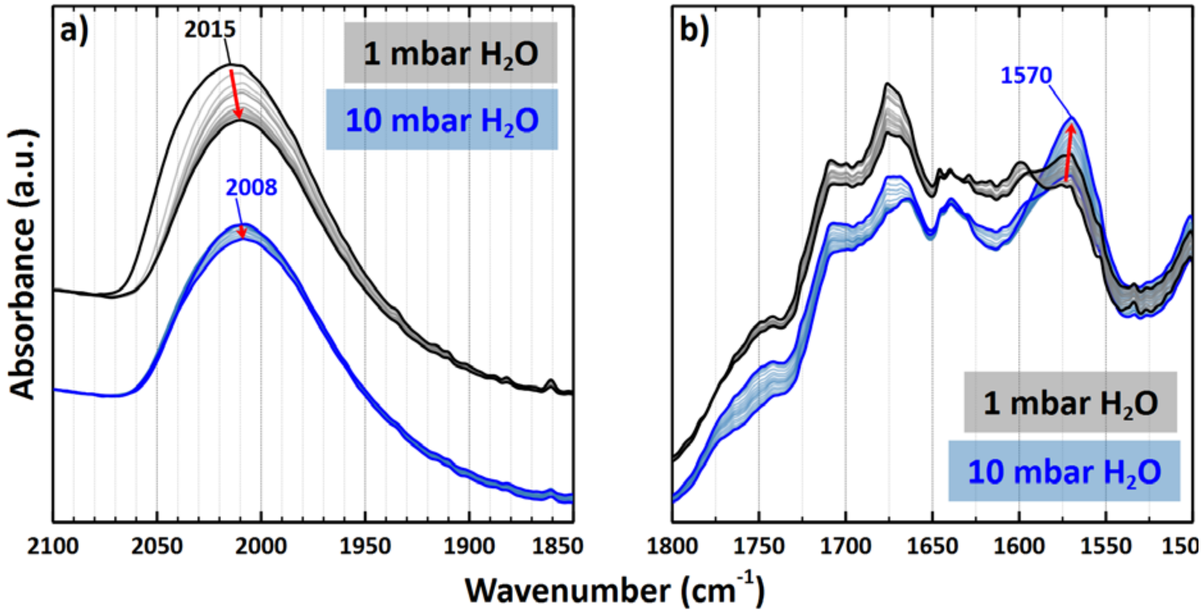

Figure S13. Attempted reforming of surface methyl groups by H_2O on 2,3 butanedione-poisoned $Pt_L/\gamma\text{-}Al_2O_3$ at 150 °C over 10 min. Black/gray and blue IR spectra represent surface species during exposure to 1 mbar H_2O vapor and further subsequent exposure to 10 mbar H_2O, respectively. Development of the **a)** CO_L and **b)** 1570 cm^{-1} ν_{as}(O-C-O) formate bands are shown.
